# Targeting the ASMase/S1P pathway protects from sortilin-evoked vascular damage in hypertension

**DOI:** 10.1172/JCI146343

**Published:** 2022-02-01

**Authors:** Paola Di Pietro, Albino Carrizzo, Eduardo Sommella, Marco Oliveti, Licia Iacoviello, Augusto Di Castelnuovo, Fausto Acernese, Antonio Damato, Massimiliano De Lucia, Fabrizio Merciai, Paola Iesu, Eleonora Venturini, Raffaele Izzo, Valentina Trimarco, Michele Ciccarelli, Giuseppe Giugliano, Roberto Carnevale, Vittoria Cammisotto, Serena Migliarino, Nicola Virtuoso, Andrea Strianese, Viviana Izzo, Pietro Campiglia, Elena Ciaglia, Bodo Levkau, Annibale A. Puca, Carmine Vecchione

**Affiliations:** 1 Department of Medicine, Surgery and Dentistry, “Scuola Medica Salernitana” University of Salerno, Baronissi, Italy.; 2 Department of Vascular Physiopathology, IRCCS Neuromed, Pozzilli, Italy.; 3 Department of Pharmacy, School of Pharmacy, University of Salerno, Fisciano, Italy.; 4 Department of Medicine and Surgery, Research Center in Epidemiology and Preventive Medicine (EPIMED), University of Insubria, Varese, Italy.; 5 Department of Epidemiology and Prevention, IRCCS Neuromed, Pozzilli, Italy.; 6 Mediterranea Cardiocentro, Naples, Italy.; 7 PhD Program in Drug Discovery and Development, University of Salerno, Fisciano, Italy.; 8 Department of Advanced Biomedical Sciences, University of Naples Federico II, Naples, Italy.; 9 Department of Neuroscience, Reproductive Sciences and Dentistry, University of Naples Federico II, Naples, Italy.; 10 Department of Medical-Surgical Sciences and Biotechnologies, Sapienza University of Rome, Latina, Italy.; 11 Department of General Surgery and Surgical Speciality Paride Stefanini, Sapienza University of Rome, Rome, Italy.; 12 Molecular and Cellular Cardiology, Department of Experimental and Clinical Medicine, Magna Graecia University, Catanzaro, Italy.; 13 European Biomedical Research Institute of Salerno (EBRIS), Salerno, Italy.; 14 Institute for Molecular Medicine III, Heinrich-Heine-University, Medical Faculty, Cardiovascular Research Institute Düsseldorf (CARID), Düsseldorf, Germany.; 15 Ageing Unit, IRCCS MultiMedica, Milan, Italy.

**Keywords:** Vascular Biology, Cardiovascular disease, Hypertension

## Abstract

Sortilin has been positively correlated with vascular disorders in humans. No study has yet evaluated the possible direct effect of sortilin on vascular function. We used pharmacological and genetic approaches coupled with study of murine and human samples to unravel the mechanisms recruited by sortilin in the vascular system. Sortilin induced endothelial dysfunction of mesenteric arteries through NADPH oxidase 2 (NOX2) isoform activation, dysfunction that was prevented by knockdown of acid sphingomyelinase (*ASMase*) or sphingosine kinase 1. In vivo, recombinant sortilin administration induced arterial hypertension in WT mice. In contrast, genetic deletion of sphingosine-1-phosphate receptor 3 (S1P3) and gp91phox/NOX2 resulted in preservation of endothelial function and blood pressure homeostasis after 14 days of systemic sortilin administration. Translating these research findings into the clinical setting, we detected elevated sortilin levels in hypertensive patients with endothelial dysfunction. Furthermore, in a population-based cohort of 270 subjects, we showed increased plasma ASMase activity and increased plasma levels of sortilin, S1P, and soluble NOX2-derived peptide (sNOX2-dp) in hypertensive subjects, and the increase was more pronounced in hypertensive subjects with uncontrolled blood pressure. Our studies reveal what we believe is a previously unrecognized role of sortilin in the impairment of vascular function and in blood pressure homeostasis and suggest the potential of sortilin and its mediators as biomarkers for the prediction of vascular dysfunction and high blood pressure.

## Graphical abstract

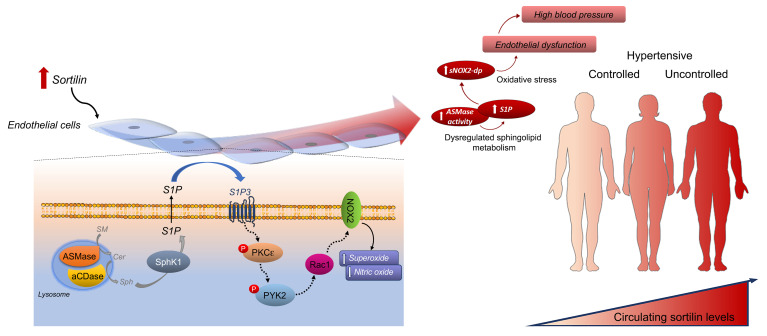

## Introduction

Over the past few years, sortilin, a member of the vacuolar protein sorting 10 (VPS10P) family of receptors, has been positively correlated with vascular and metabolic disorders (1). Preclinical in vivo evidence suggested that sortilin promotes insulin resistance in type 2 diabetes mellitus and dyslipidemia by altering hepatic apolipoprotein B-100 metabolism (2, 3). Sortilin has been also implicated in vascular calcification and inflammation, acting by favoring the development of arterial atherosclerosis (4, 5). Moreover, circulating sortilin levels were found associated with higher risk of major adverse cerebrovascular and cardiovascular events (MACCE), coronary artery disease and peripheral arterial disease (PAD) (6–8). To date, the cardiovascular effects of sortilin have been mostly linked to altered circulating cholesterol levels (9–12). However, growing evidence suggests that sortilin may contribute to the pathogenesis of cardiovascular diseases independently of its role in lipid metabolism (4–6), pointing to the existence of alternative mechanisms of action.

Although preclinical and clinical investigations have highlighted the critical role of sortilin in the pathogenesis of vascular disorders, so far, no studies have investigated a possible direct role of sortilin in the modulation of vascular function. Here, we demonstrate that (a) sortilin induces excessive generation of ROS through the activation of NADPH oxidase (NOX); (b) sortilin evokes endothelial dysfunction in mouse mesenteric arteries via acid sphingomyelinase–dependent (ASMase-dependent) dysregulation of sphingosine-1-phosphate (S1P) signaling, leading to the activation of the NOX2 isoform; (c) in vivo, sortilin administration induces arterial hypertension, an effect not seen in mice lacking S1P lysosphingolipid receptor 3 or gp91^phox^/NOX2; (d) circulating sortilin is elevated in hypertensive individuals with endothelial dysfunction; (e) plasma ASMase activity and plasma levels of sortilin, S1P, and soluble NOX2-derived peptide (sNOX2-dp) are higher in hypertensive than in normotensive subjects, but even higher in uncontrolled hypertensive ones; and( f) circulating sortilin levels are directly correlated with S1P levels in hypertensive patients.

## Results

### Sortilin induces endothelial dysfunction through increased oxidative stress.

**Figure 1:**
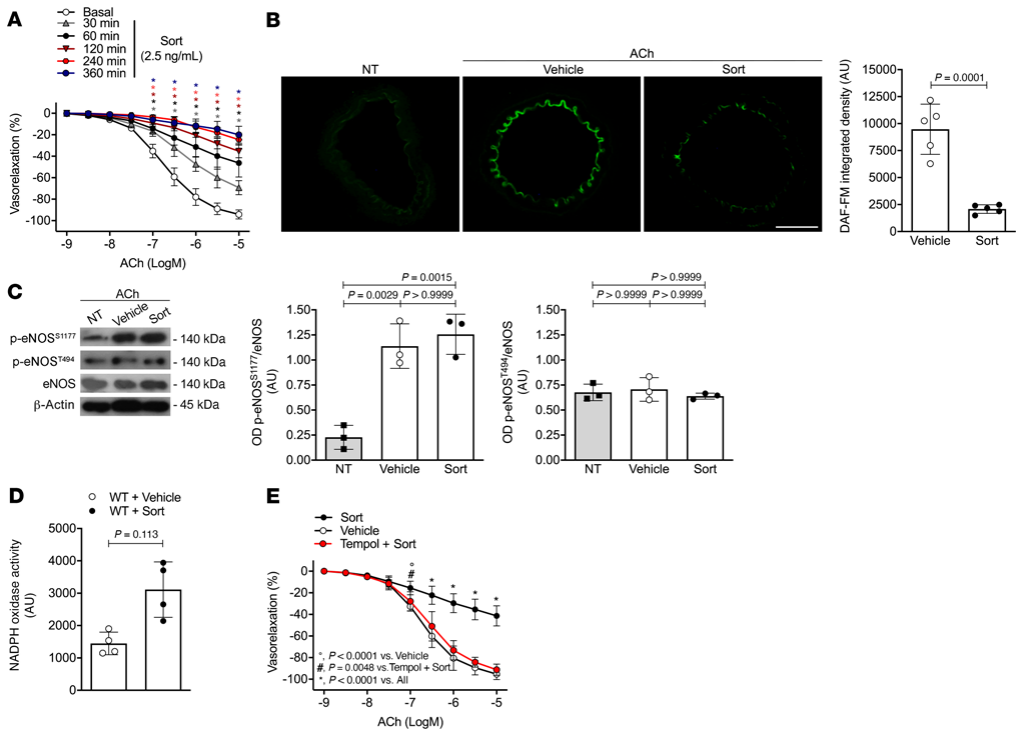
Sortilin impairs endothelium-dependent vasorelaxation through increased ROS production. (**A**) Acetylcholine-evoked vasorelaxation in WT mesenteric arteries exposed to vehicle or sortilin (Sort) at 2.5 ng/mL for different preincubation times (30, 60, 120, 240, and 360 minutes) (*n* = 6). ACh, acetylcholine. (**B**) Detection of NO by DAF-FM fluorescence in untreated WT mesenteric arteries (NT) stimulated with acetylcholine alone (10^–5^ M) (vehicle) or after pretreatment with sortilin. Scale bar: 50 μm. Bar graph showing DAF-FM fluorescence integrated density in the endothelial layer. A.U., arbitrary units of fluorescence (*n* = 5). (**C**) Representative immunoblots and densitometric analyses of 3 independent experiments evaluating protein levels of phospho-Ser1177-eNOS, phospho-Thr494-eNOS, and total eNOS expression in untreated WT mesenteric arteries (NT) stimulated with acetylcholine alone (10^–5^ M) (vehicle) or after pretreatment with sortilin for 60 minutes. (**D**) Effect of sortilin on NOX activity in WT mesenteric arteries. Data are expressed as increase of chemiluminescence per minute in arbitrary units (*n* = 4). (**E**) Acetylcholine-evoked vasorelaxation in WT mesenteric arteries exposed to vehicle, sortilin alone, or pretreated with tempol for 30 minutes prior to sortilin (*n* = 6). Data are represented as mean ± SD. Statistical analysis was determined by unpaired 2-tailed Student’s *t* test (**B** and **D**); 1-way ANOVA (**C**); or 2-way ANOVA followed by Bonferroni’s post hoc test (**A** and **E**). (**A**) *P < 0.0001 versus basal at the same acetylcholine concentration (as indicated by color code). (**E**) °*P* < 0.0001 versus Vehicle. ^#^*P* = 0.0048 versus Tempol + Sort. *P < 0.0001 versus All.

Since an elevated plasma sortilin level has been associated with a high prevalence of cardiovascular risk factors in patients (4, 13, 14), we first aimed to investigate whether the protein per se was able to influence vascular function. In vascular relaxation studies, sortilin induced a time- and dose-dependent impairment of vasodilator response to acetylcholine in mouse mesenteric vessels (Figure 1A and Supplemental Figure 1, A and B; supplemental material available online with this article; https://doi.org/10.1172/JCI146343DS1). As we noted a prominent impairment in vascular reactivity already after 60 minutes of sortilin preincubation with 2.5 ng/mL, we decided to use this experimental setting for all subsequent in vitro experiments. Sortilin impaired neither smooth muscle vasorelaxation evoked by nitroglycerin nor the vasoconstrictive response induced by the thromboxane A2 receptor agonist U46619 (Supplemental Figure 1, C and D), which suggests that likely the endothelium is the target of sortilin’s vascular action. Nitric oxide (NO) is the most important endothelial-derived factor responsible for the maintenance of vascular function (15). In isolated mesenteric arteries, sortilin caused a marked reduction of NO levels (Figure 1B) without affecting phosphorylation of endothelial NO synthase (eNOS) at both Ser1177 and Thr494 (Figure 1C), positive and negative regulatory sites of the enzyme, respectively (16). In addition, the increase in ROS production and the finding that endothelial dysfunction was almost entirely prevented by pretreatment with the free-radical scavenger tempol (Figure 1, D and E) clearly suggested oxidative stress as the major determinant of reduced NO bioavailability in response to sortilin.

### Sortilin induces endothelial dysfunction by altering sphingolipid metabolism.

**Figure 2:**
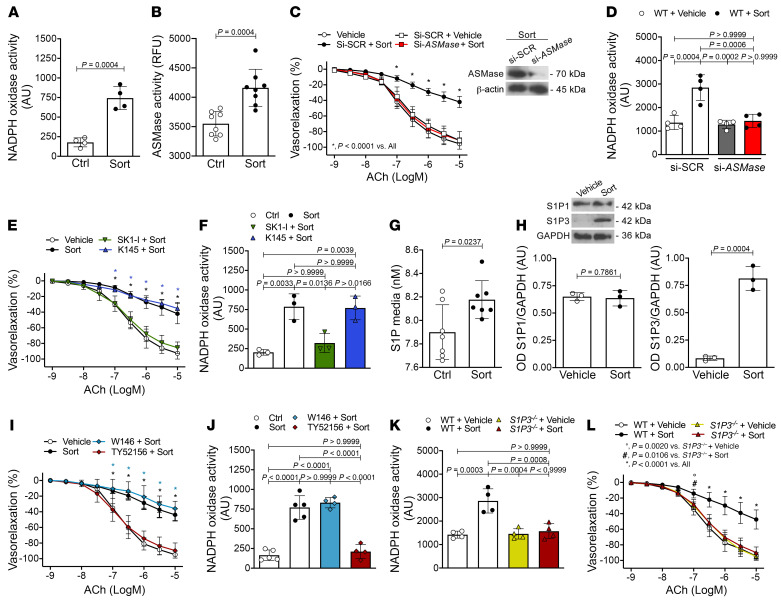
Sortilin evokes endothelial dysfunction through the S1P-signaling pathway. (**A**) NOX activity (*n* = 4) and (**B**) ASMase activity (*n* = 8) in HUVECs treated with vehicle (Ctrl) or sortilin. RFU, relative fluorescence units. (**C**) Acetylcholine-evoked vasorelaxation in WT mesenteric arteries exposed to vehicle or pretransfected with siRNA against *ASMase* (Si-*ASMase*) before sortilin; scrambled siRNA (Si-SCR) was used as control (*n* = 6). ASMase protein levels after siRNA silencing. (**D**) Effect of si-*ASMase* on NOX activity in WT mesenteric arteries exposed to sortilin (*n* = 4). (**E**) Acetylcholine-evoked vasorelaxation in WT mesenteric arteries exposed to vehicle or sortilin or pretreated with either K145 or SK1-I before sortilin (*n* = 5). (**F**) Effects of K145 and SK1-I on NOX activity in sortilin-stimulated HUVECs (*n* = 3). (**G**) LC-MS/MS quantification of extracellular S1P levels in vehicle- or sortilin-treated HUVECs (*n* = 7). (**H**) Representative immunoblots and densitometric analyses of 3 independent experiments evaluating S1P1 and S1P3 expression in WT mesenteric arteries exposed to vehicle or sortilin. (**I**) Acetylcholine-evoked vasorelaxation in WT mesenteric arteries exposed to vehicle or sortilin or pretreated with W146 or TY52156 before sortilin (*n* = 6). (**J** and **K**) NOX activity in (**J**) HUVECs treated with vehicle or sortilin or pretreated with W146 or TY52156 and in (**K**) *S1P3^–/–^* mesenteric arteries exposed to vehicle or sortilin (*n* = 4–5 replicates from 3 independent experiments). (**L**) Acetylcholine-evoked vasorelaxation in mesenteric arteries from WT and *S1P3^–/–^* mice exposed to vehicle or sortilin (*n* = 5). Data are represented as mean ± SD. Unpaired Student’s *t* test (**A**, **B**, **G**, and **H**); 1-way (**D**, **F**, **J**, and **K**) or 2-way (**C**, **E**, **I**, and **L**) ANOVA followed by Bonferroni’s post hoc test. *P < 0.0001 versus vehicle or SK1-I plus sortilin at the same acetylcholine concentration (as indicated by color code) (**E**); *P < 0.0001 versus vehicle or TY52156 plus sortilin at the same acetylcholine concentration (as indicated by color code) (**I**).

ASMase is a lipid hydrolase that cleaves sphingomyelin to produce bioactive sphingolipids. Previous mechanistic studies have reported that, in response to Fas stimulation, sortilin promotes trafficking and exposure of lysosome-targeted ASMase to the cell-membrane surface, a process leading to lipid raft clustering and assembly of NOX (17, 18). In HUVECs, we show that sortilin evoked, per se, NOX activation and increased intracellular ASMase activity (Figure 2, A and B). siRNA-mediated knockdown of *ASMase* effectively protected against endothelial dysfunction as well as NADPH-derived ROS overproduction in mesenteric arteries as compared with scrambled siRNA after sortilin exposure (Figure 2, C and D), thus unravelling the critical role played by ASMase in the vascular effects of sortilin. Ceramide is the immediate metabolic product of ASMase action, which can be further modified to form complex sphingolipids or broken down into sphingosine and free fatty acids by ceramidases (19). We next investigated whether sphingosine or its precursor ceramide mediates the deleterious effect of sortilin. Knockdown of the acid ceramidase (*aCDase*), responsible for sphingosine generation by cleavage, effectively protected from sortilin-evoked endothelial dysfunction (Supplemental Figure 2A), thus demonstrating that sphingosine, but not ceramide, is responsible for the endothelial injury induced by sortilin. To determine whether sphingosine or its phosphorylated derivative S1P could be accountable for the sortilin-dependent NOX activation pathway, we investigated the potential contribution of sphingosine kinase 1 (SphK1) and SphK2, which generate the endogenous mediator S1P. Interestingly, only pharmacological inhibition of SphK1 with SK1-I or its genetic suppression protected against sortilin-induced endothelial dysfunction and superoxide generation in HUVECs, whereas these effects were not observed after inhibition of SphK2 with either K145 or siRNA-mediated knockdown (Figure 2, E and F, and Supplemental Figure 2, B and C). Remarkably, quantification of sphingolipid levels by liquid chromatography–tandem mass spectrometry (LC-MS/MS) in sortilin-treated endothelial cells showed a consistent decrease in intracellular ceramide content, including that of cer-C16, cer-C18, cer-C22, cer-C24, and cer-C24:1 (Supplemental Figure 2D), that was paralleled by increased extracellular S1P levels (Figure 2G). This indicates that sortilin drives the activation of the ASMase/aCDase pathway, leading to an altered rheostat in favor of S1P at the expense of ceramide. We then explored whether depletion of ceramides rather than accumulation of S1P is responsible for endothelial dysfunction caused by sortilin. Our results showed that neither C16 nor C18 ceramide pretreatment (10 nM) was able to prevent sortilin vascular effect, while pretreatment with *aCDase* siRNA protected from endothelial dysfunction induced by sortilin (Supplemental Figure 2, E and F). Besides corroborating a dysregulated sphingolipid signaling in the endothelium, these data suggest that strategies aimed at restoring ceramide levels are ineffective in preventing the endothelial dysfunction evoked by sortilin unless coupled with blocking of the generation of sphingosine and its derivative active form.

### Pharmacological and genetic inhibition of the S1P3 receptor prevents sortilin-induced endothelial dysfunction and vascular oxidative stress.

Given the importance of S1P1 and S1P3 in the regulation of the vascular tone of resistance vessels (20), we next investigated their role in the sortilin signaling pathway. Sortilin caused an upregulation of S1P3, but not S1P1, expression, in WT mesenteric arteries (Figure 2H). In HUVECs, flow cytometry analysis revealed that sortilin induced a substantial increase in both the percentages of cells expressing the receptor (percentages of positive cells) and the amount of S1P3 expression after 30 minutes of stimulation, whereas at later time points, a downregulation of the receptor (both in terms of percentage and amount) was observed (Supplemental Figure 3, A and B). Measurement of mRNA relative expression of *S1P3* at different time points revealed that sortilin induced a progressive increase of S1P3 de novo synthesis, starting at 60 minutes and for up to 360 minutes (Supplemental Figure 3C). These results may explain the time-dependent effect of sortilin on vascular function. In detail, via S1P, sortilin induced S1P3 activation through a first round of mobilization of the reserve pool of receptors, followed by induction of the de novo synthesis pool in a time-dependent manner. We then evaluated vascular endothelial–cadherin (VE-cadherin) expression following sortilin exposure in vitro, being that it is the principal protein regulating formation of adherens junctions and barrier permeability in endothelial cells. Immunofluorescent staining against VE-cadherin showed the loss of endothelial cell-cell contacts in HUVECs exposed to sortilin for 1 hour, an effect blunted by S1P3 pharmacological inhibitor TY52156 (Supplemental Figure 4). Inhibition of S1P3 with TY52156 or gene silencing prevented sortilin-induced endothelial dysfunction as well as oxidative stress in mesenteric arteries and HUVECs, whereas these effects were not observed after S1P1 inhibition with W146 or siRNA-mediated knockdown (Figure 2, I and J, Supplemental Figure 5, A and B, and Supplemental Figure 6, A and B). Consistently, mesenteric arteries from S1P3-deficient mice were completely protected against sortilin-induced ROS production and endothelial dysfunction (Figure 2, K and L), clearly supporting the thesis that S1P3 mediates the crosstalk between S1P and NOX and sortilin’s deleterious effects.

### Sortilin induces vascular oxidative stress by promoting Rac1-driven activation of NOX2.

**Figure 3:**
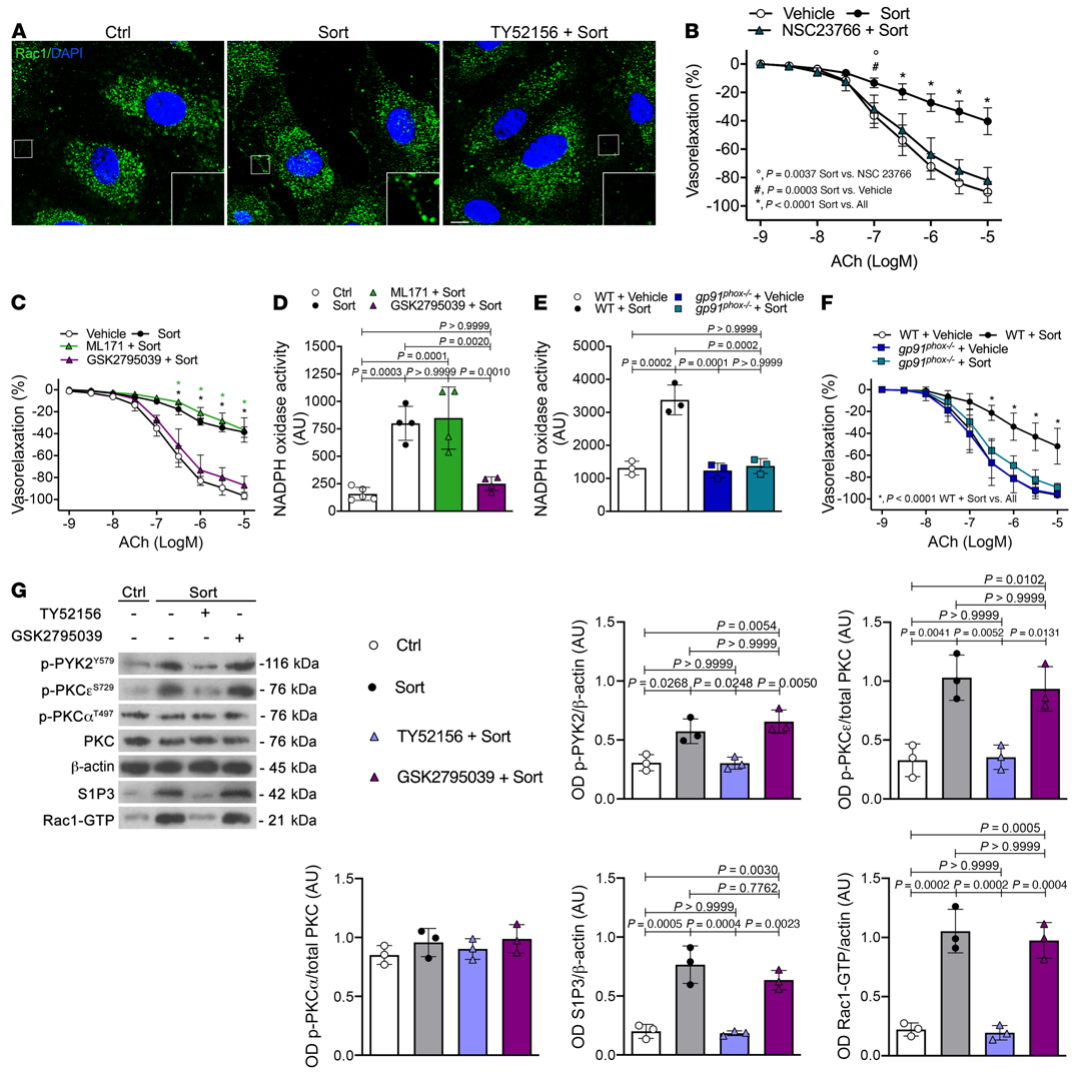
Phosphorylation of PKCɛ and PYK2 is required for Rac1-dependent NOX2 activation. (**A**) Representative confocal immunofluorescence staining of Rac1 in HUVECs treated with vehicle (Ctrl) or sortilin alone or pretreated with the S1P3 inhibitor TY52156. Arrows indicate membrane translocation of Rac1. Scale bar: 10 μm. Insets show higher magnification, zoom ×4.7. (**B**) Acetylcholine-evoked vasorelaxation in WT mesenteric arteries exposed to vehicle, sortilin alone, or sortilin in the presence of NSC23766 (*n* = 3). (**C**) Acetylcholine-evoked vasorelaxation in WT mesenteric arteries treated with vehicle or sortilin alone or pretreated with either ML171 or GSK2795039 before sortilin stimulation (*n* = 3). (**D**) NOX activity in HUVECs treated with vehicle or sortilin alone or preincubated with ML171 or GSK2795039 before sortilin (*n* = 4–5 replicates from 3 independent experiments). (**E**) NOX activity in WT and *gp91^phox–/–^* mesenteric arteries exposed to vehicle or sortilin (*n* = 3 replicates from 3 independent experiments). Data are expressed as increase of chemiluminescence per minute in arbitrary units. (**F**) Acetylcholine-evoked vasorelaxation in mesenteric arteries from WT and *gp91^phox–/–^* mice exposed to vehicle or sortilin (*n* = 5). (**G**) Representative immunoblots and densitometric analyses of 3 independent experiments evaluating protein levels of phospho-Tyr579-PYK2, phospho-Ser729-PKCɛ, phospho-Thr497-PKCα, PKC, S1P3, and Rac1-GTP in HUVECs treated with vehicle or sortilin in the presence or absence of TY52156 or GSK2795039. Data are represented as mean ± SD. One-way ANOVA (**D**, **E**, and **G**) or 2-way ANOVA (**B**, **C**, and **F**) followed by Bonferroni’s post hoc test was used. (**C**) *P < 0.0001 versus vehicle or GSK2795039 plus sortilin at the same acetylcholine concentration (as indicated by color code).

Upon activation, the NOX complex undergoes posttranslational modifications of its oxidase regulatory subunits, allowing association with the membrane catalytic subunit. Also, some NOX isoforms require a small GTPase (Rac1 or Rac2) for their activation (21). In endothelial cells, we found that sortilin promoted Rac1 activation, as demonstrated by translocation of the latter to the plasma membrane, an effect prevented by pretreatment with the S1P3 inhibitor TY52156 (Figure 3A). Of note, the use of the Rac1 inhibitor NSC23766 was able to prevent the impaired dilator response to acetylcholine evoked by sortilin (Figure 3B). We next determined the individual contribution of different NOX isoforms in sortilin-induced vascular oxidative stress. NOX2 inhibition by GSK2795039 effectively prevented endothelial dysfunction as well as oxidative stress induced by sortilin, whereas these effects were not observed with the NOX1 inhibitor ML171 (Figure 3, C and D). Exposure of HUVECs to sortilin enhanced mRNA expression of *NOX2*, which was prevented by the S1P3 pharmacological inhibitor TY52156 (Supplemental Figure 7A). In contrast, no differences of *NOX4* at mRNA levels were observed (Supplemental Figure 7B). Sortilin-induced superoxide generation and NO reduction were prevented by both superoxide dismutase–polyethylene glycol (PEG-SOD), a potent scavenger of oxygen free radicals, and gp91 ds-tat, a specific peptide that blocks NOX2 assembly and activation (Supplemental Figure 8, A–D). Remarkably, mesenteric arteries from NOX2-deficient mice (*gp91*^phox–/–^) were significantly protected against sortilin-induced NOX activation and endothelial dysfunction (Figure 3, E and F). Overall, these results clearly indicate NOX2 as the effector of sortilin-mediated vascular oxidative stress via the ASMase/S1P/S1P3 axis.

### PKCɛ acts as a downstream modulator of S1P3 to mediate sortilin-induced vascular injury.

To define the mechanism by which the interaction of sortilin and S1P3 activates the Rac-1 machinery, we investigated the potential involvement of 2 PKC isoforms and proline-rich tyrosine kinase 2 (PYK2), which have been previously implicated in ROS production (22–24).

**Figure 4:**
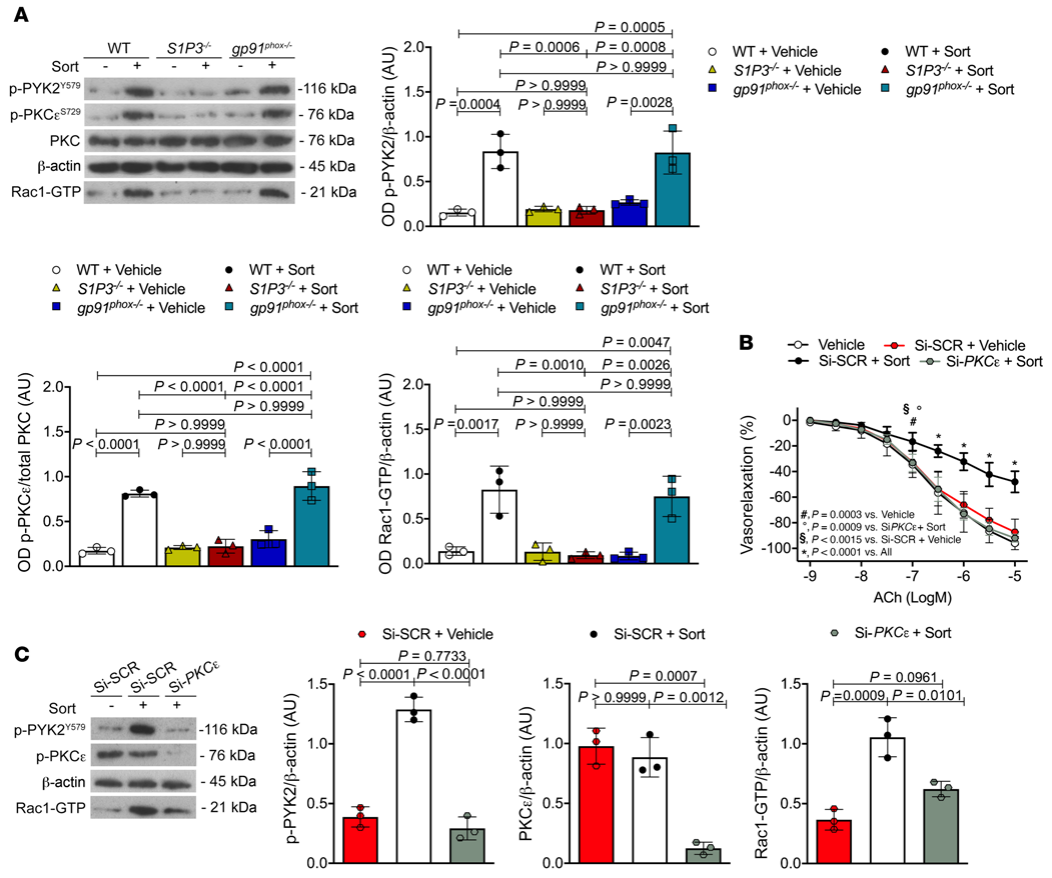
PKCɛ is involved in sortilin-induced vascular damage. (**A**) Representative immunoblots and densitometric analyses of 3 independent experiments evaluating protein levels of phospho-PYK2, phospho-PKCɛ, and Rac1-GTP in WT, *S1P3^–/–^*, and *gp91^phox–/–^* vessels exposed to vehicle or sortilin for 60 minutes. (**B**) Acetylcholine-evoked vasorelaxation in WT mesenteric arteries exposed to vehicle or pretransfected with either siRNA silencing *PKC*ɛ or a scrambled siRNA and then exposed to sortilin for 60 minutes (*n* = 6). (**C**) Representative immunoblots and densitometric analyses of 3 independent experiments evaluating phospho-PYK2, and Rac1-GTP levels in WT mesenteric arteries pretransfected with either siRNA silencing *PKC*ɛ or a scrambled siRNA and then exposed to sortilin; the effectiveness of *PKC*ɛ silencing was determined by Western blotting (*n* = 3). Data are represented as mean ± SD. One-way ANOVA (**A** and **C**) or 2-way ANOVA (**B**) followed by Bonferroni’s post hoc test was used.

In HUVECs, sortilin did not cause activation of PKCα (T497), while it increased phosphorylation of PKCɛ (S729) and PYK2 (Y579) (Figure 3G). Selective inhibition of S1P3 with TY52156 prevented activation of PKCɛ, PYK2, and Rac1-GTP, whereas pretreatment with NOX2 inhibitor GSK2795039 did not (Figure 3G). Consistent with this, sortilin induced phosphorylation of PKCɛ and PYK2 in mesenteric arteries from WT and *gp91^phox–/–^* mice, but not from S1P3-deficient mice (Figure 4A). Of note, knockdown of *PKC*ɛ abolished sortilin-induced endothelial dysfunction in WT mouse mesenteric arteries (Figure 4B) as well as activation of PYK2 and Rac1 (Figure 4C). These findings strongly support the notion that PKCɛ participates in sortilin signaling, acting as a downstream effector of S1P3 and an upstream modulator of PYK2 and Rac1.

### S1P3 or gp91^phox^ deficiency protects from arterial hypertension and endothelial dysfunction evoked in vivo by sortilin administration.

Oxidative stress and endothelial dysfunction are strongly associated with hypertension (25–27). Based on the above findings, we assessed the effects of sortilin on blood pressure (BP) in vivo. A single administration of recombinant sortilin (40 ng/mL, i.p.) induced a transient increase of BP in WT mice; the effect persisted for 4 hours, with near-baseline levels returning within 6 hours of treatment (Supplemental Figure 9A). When we excised vessels for vascular reactivity studies, we observed endothelial dysfunction in WT vessels (Supplemental Figure 9B). In contrast, administration of sortilin did not promote BP increase and vascular dysfunction in either S1P3- or gp91^phox^-deficient mice (Supplemental Figure 9, C–F). Notably, a single injection of the S1P3 inhibitor TY52156 one hour from sortilin administration was sufficient to rescue the harmful effect, lowering BP to normal values in WT mice (Supplemental Figure 9G). Western blot analysis confirmed the loss of gp91^phox^ and S1P3 protein expression in mesenteric arteries of both strains of mice (Supplemental Figure 9H).

**Figure 5:**
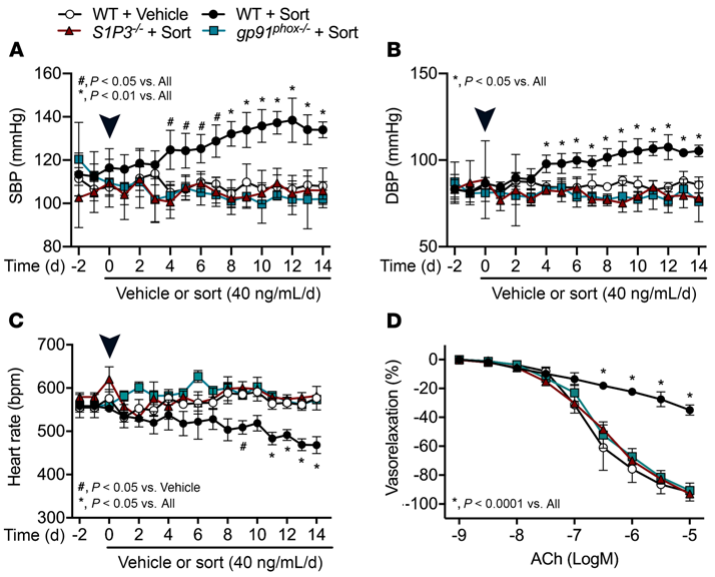
Genetic deletion of either *S1P3* or *gp91^phox^* protects mice from chronic sortilin-induced high BP and endothelial dysfunction. (**A**–**C**) SBP, DBP, and heart rate determined by telemetry before and after implantation of an osmotic pump delivering vehicle or sortilin in WT, *S1P3^–/–^*, and *gp91^phox–/–^* mice (*n* = 4). Arrowheads indicate the day of implantation. (**D**) Acetylcholine-evoked vasorelaxation in mesenteric arteries from WT, *S1P3^–/–^*, and *gp91^phox–/–^* mice treated with vehicle or sortilin with an osmotic pump for 14 days (*n* = 4). Data are represented as mean ± SD. Two-way ANOVA (**D**) or 2-way ANOVA repeated measures (RM) (**A**–**C**) followed by Bonferroni’s post hoc test was used.

To mimic an in vivo condition of sustained high circulating levels of sortilin, mice were implanted with osmotic pumps delivering recombinant sortilin protein for 14 days. According to the literature (20, 28), genetic deletion of *S1P3* or *gp91^phox^* does not influence BP levels under basal conditions (Supplemental Figure 10A). Chronic infusion of sortilin caused a sustained increase in arterial BP and a decrease in heart rate in WT mice (Figure 5, A–C, and Supplemental Figure 10B). It is noteworthy that the evaluation of vascular function in mesenteric arteries excised after 14 days of in vivo sortilin treatment revealed there was an impairment of endothelial-dependent vasorelaxation in WT mouse vessels (Figure 5D) that was similar to that observed in the experiments in which sortilin was administered as a single dose or ex vivo. Even in this chronic paradigm of administration, either S1P3- or gp91^phox^-deficient mice were completely protected from sortilin-evoked BP increase, heart rate decrease, and endothelial vasorelaxation impairment (Figure 5, A–C, and Supplemental Figure 10, C and D). Furthermore, sortilin treatment enhanced oxidative stress in plasma and kidney, vascular permeability, and peroxynitrite formation and infiltration of inflammatory cells in mesenteric arteries (Supplemental Figures 11 and 12). Besides demonstrating the critical involvement of S1P3 and NOX2 in sortilin-mediated high BP, these results also support the hypothesis of a causal role for sortilin-dependent endothelial dysfunction in the development of hypertension.

### Hypertensive patients with endothelial dysfunction have elevated plasma levels of sortilin.

**Figure 6:**
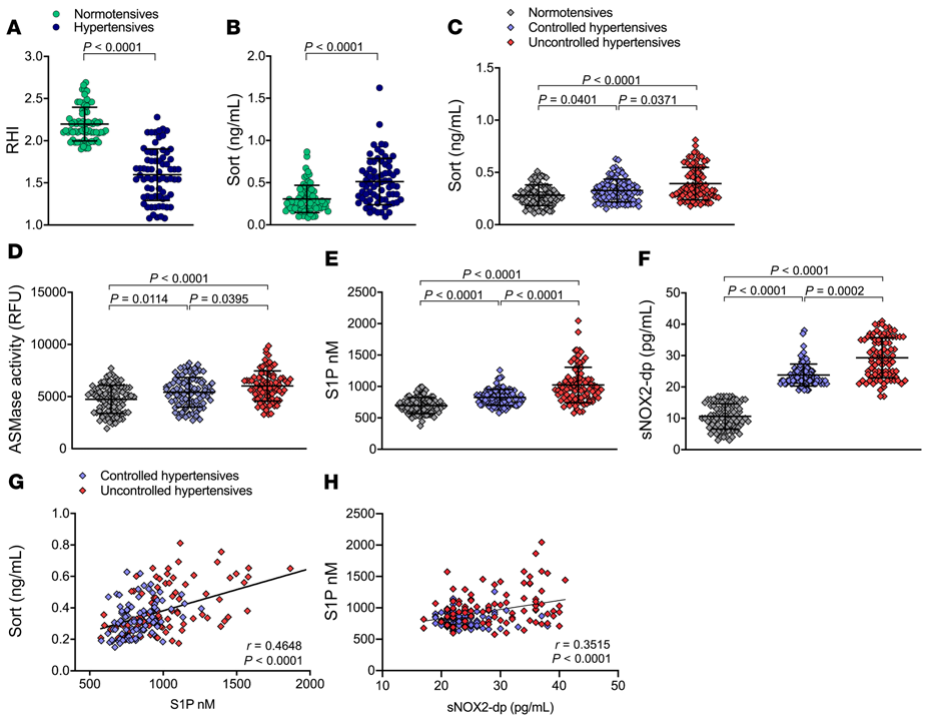
Sortilin levels are elevated in hypertensive patients with endothelial dysfunction, sphingolipid dysregulation, and oxidative stress. (**A**) RHI and (**B**) plasma sortilin levels in normotensive and hypertensive subjects from the Campania Salute Network (*n* = 71 normotensive; *n* = 71 hypertensive). Plasma levels of (**C**) sortilin, (**D**) ASMase activity (RFU), (**E**) S1P, and (**F**) sNOX2-dp in normotensive patients and controlled and uncontrolled hypertensive patients from the Moli-sani Study (*n* = 81 normotensive, *n* = 91 controlled hypertensive, *n* = 90 uncontrolled hypertensive). (**G**) Pearson’s correlation coefficient analysis between sortilin and S1P plasma levels in the entire hypertensive population. (**H**) Pearson’s correlation coefficient analysis between S1P and sNOX2-dp plasma levels in the entire hypertensive population. Data are represented as mean ± SD. Unpaired Student’s *t* test with Welch’s correction (**A** and **B**); nonparametric Kruskal–Wallis test with Dunn’s correction (**C**–**F**).

To translate our experimental findings, we first investigated plasma sortilin concentrations in hypertensive patients belonging to the Campania Salute Network who had undergone endothelial function evaluation. Characteristics of the study population are presented in Table 1. As published before (29) and shown in our cohort, hypertensive patients showed lower mean reactive hyperemia index (RHI) compared with healthy subjects (Figure 6A). Interestingly, plasma levels of sortilin were substantially increased in hypertensive patients when compared with normotensive individuals (Figure 6B), thus supporting the relevance of sortilin for endothelial dysfunction in patients with arterial hypertension.

Hypertension is the major cardiovascular risk factor for the development and progression of PAD 30). Since sortilin was significantly associated with the presence of PAD (8), to exclude the potential influence of this comorbidity in our cohort of patients, we replicated our data in an independent cohort of hypertensive individuals with no previous history of PAD symptoms or confirmed PAD diagnosis. Clinical characteristics are summarized in Supplemental Table 1. Even in this cohort of patients, the evaluation of circulating sortilin levels showed significant elevation in hypertensive individuals without PAD as compared with the control group (Supplemental Figure 13). These data fully support the thesis that sortilin is involved in the pathogenesis or sequelae of hypertension independently of its association with PAD.

### Sortilin levels are associated with dysregulated sphingolipid metabolism and oxidative stress in humans with arterial hypertension.

To strengthen the translational relevance of our findings, we then extended our studies to measure plasma levels of sortilin, S1P, and sNOX2-dp and ASMase activity in a large population-based cohort of the Moli-sani study. This cohort consisted of hypertensive patients taking antihypertensive medication who were stratified into controlled hypertensive and uncontrolled hypertensive as well as normotensive control subjects. The clinical and biochemical characteristics are summarized in Table 2. The 3 groups were strictly matched with respect to age, sex, and laboratory characteristics, but differed substantially in terms of BP levels. Plasma sortilin levels, ASMase activity, and S1P and sNOX2-dp levels were markedly elevated in the entire hypertensive population as compared with healthy controls, and the increase was even more pronounced in hypertensive patients with uncontrolled BP than in controlled hypertensive and normotensive counterparts (Figure 6, C–F). ANOVA excluded a potential influence of antihypertensive drug therapy on plasma sortilin concentrations (Supplemental Table 2). Notably, a significant positive correlation was found between circulating sortilin and S1P levels in the entire hypertensive population (*r* = 0.465, *P* < 0.0001; Figure 6G). We also observed a positive correlation between S1P and sNOX2-dp plasma levels in the same samples (*r* = 0.3515; *P* < 0.0001; Figure 6H).

## Discussion

We demonstrate here for what we believe is the first time the direct role that sortilin has on vascular function. In particular, we found that sortilin promotes endothelial dysfunction and arterial hypertension in mice via a sphingolipid-dependent mechanism. Additionally, we provide evidence supporting the notion that sortilin plays a pivotal role in dysfunctional sphingolipid metabolism and oxidative stress associated with hypertension in humans.

Previous work has shown some mechanistic insight into the role of sortilin in mediating NOX activation (18). Using an in vitro model, Bao et al. (18) demonstrated that, in response to Fas receptor stimulation, sortilin together with ASMase promotes the trafficking of lysosomes toward the cell membranes, resulting in clustering of lipid rafts and consequent aggregation of NADPH subunits. Moreover, the decrease in NO levels induced by FasL in isolated bovine coronary arteries was efficiently reversed by sortilin inhibition, an effect similar to that observed with NOX pharmacological inactivation (18), thus suggesting a key role of sortilin in the ceramide/redox signaling pathway in endothelial cells.

In the present study, sortilin-induced ROS overproduction and the consequential endothelial dysfunction of resistance vessels were completely prevented by knockdown of *ASMase*, suggesting that circulating sortilin, per se, produces a deleterious vascular effect by directly promoting ASMase activation. Once activated, the ASMase, translocated to the outer cell membrane, gives rise to ceramide production that, via the activity of SphKs, can be further metabolized into S1P (31). Owing to the interconvertible nature of these bioactive sphingolipids, the fine balance between antiproliferative activity of ceramide and antiapoptotic effects of S1P, also referred to as sphingolipid rheostat, is critical for cellular homeostasis (32). Even though studies conducted over the last decade substantially extended our knowledge in the context of sphingolipids’ origin and metabolism, many fundamental questions about the mechanisms underlying both their homeostasis and dysregulation remain to be solved. In addition to red blood cells, endothelial cells actively produce and release S1P into the bloodstream, thus representing an additional important source of plasma S1P (33). Its effects are mostly mediated by the activation of 5 high-affinity G protein–coupled receptors, namely S1P1–5 (34), whose cellular and temporal expression have been proposed as critical determinants for their specific roles in various organ systems. Among these, S1P1, S1P2, and S1P3 receptors are the major subtypes in the cardiovascular system (35). Although previous studies have shown that S1P exerts vasculoprotective effects (20, 36), our data provide strong evidence that, in response to sortilin, ASMase-mediated NOX activation requires the action of S1P. It is important to underline that, unlike previous studies evaluating in vitro or ex vivo vascular cell responses following exogenous S1P stimulation (20, 37), here, we examined the effects evoked by the modulation of endogenous S1P levels. Thus, in our experimental conditions, the adverse effects of S1P on endothelial cells may be due to the alternative sphingolipid metabolic pathway dependent on ASMase activation that results in an altered rheostat in favor of S1P at the expense of ceramide. Through a pharmacological approach, we identified S1P3 as the key S1P receptor mediating the vascular effects evoked by sortilin. In agreement, S1P3-deficient vessels were completely protected against sortilin-induced endothelial dysfunction and ROS overproduction.

It is generally accepted that Rac1 plays a critical role in activation of the NOX complex. Herein, we also established the recruitment of Rac1 into the signaling cascade leading to ROS generation in response to sortilin, an effect mediated by NOX2, since its deficiency prevented sortilin-induced ROS production as well as endothelial dysfunction in mouse mesenteric resistance arteries.

PKC is a critical player that contributes to the regulation of the vascular system under both physiological and pathophysiological conditions (38). Previous works have indicated PKC activation as a relevant pathway responsible for NOX activation in vascular tissues (24, 39). Of interest, Lin and colleagues (23) demonstrated the ability of S1P to induce ROS generation via direct cooperation between PKC and PYK2. Among PKC isoforms, both PKCα and PKC have been reported as participating in PYK2 activation (40, 41). These reports led us to investigate the intracellular signaling events between S1P3- and NOX2-dependent ROS production, focusing especially on the PKC pathway. In isolated endothelial cells, sortilin caused activation of PKCɛ and PYK2, an effect blunted by TY52156 pretreatment, but not with the NOX2 inhibitor. These findings were strongly supported by the fact that, in contrast with sortilin-treated WT and *gp91^phox–/–^* vessels, the effect was absent in *S1P3^–/–^* vessels, an observation clearly identifying, on the one hand, PKCɛ and PYK2 as downstream effectors of S1P3 and upstream regulators of the Rac1/NOX2 pathway on the other. Taken together, our results have unmasked a molecular mechanism underpinning the role of sortilin in sphingolipid dysregulation, oxidative stress, and endothelial dysfunction in the pathophysiology of vascular complications.

Although endothelial dysfunction has long been considered as a detrimental effect of high BP, there is now compelling evidence that vascular oxidative stress has a causal role in the pathogenesis of hypertension (25–27). In this regard, studies in human resistance vessels transfected with *gp91^phox^* antisense oligonucleotides (42) as well as in endothelial cell–specific *NOX2*-knockout mice (43) have demonstrated the undeniable contribution of NOX2-derived oxidative stress to the development of hypertension.

Since mesenteric arteries represent the prototype of resistance vessels for BP regulation, we investigated the effects of sortilin in acute and chronic administration paradigms in vivo. Sortilin caused increased BP in WT mice, a hemodynamic effect that was entirely counteracted by deficiency of S1P3 or gp91^phox^. While acute administration resulted in a transient increase in BP in WT mice, chronic sortilin infusion induced sustained arterial pressure elevation over all the observation period from the third day on. Notably, in both experimental settings, in vivo administration of recombinant sortilin induced endothelial dysfunction, whereas the lack of S1P3 or gp91^phox^ resulted in a strong protective effect against vascular dysfunction. An interesting observation of this study is that the impaired endothelium-dependent relaxation observed with sortilin after in vivo administration was similar to that obtained in vitro, a setting that cannot be influenced by the systemic inflammatory response or sympathetic activation. Thus, these results strongly support the notion that activation of the S1P/NOX2 signaling axis induced by sortilin and the subsequent impairment of endothelial-dependent dilation may be causally involved in the increased BP detected after in vivo administration of the protein.

Translating our experimental studies to humans, we found that circulating sortilin levels were elevated in hypertensive individuals with impaired endothelial function, thus supporting the hypothesis of its involvement in the pathogenesis or sequelae of hypertension. This finding was further evaluated in a second cohort of individuals without additional comorbidities and stratified by BP level. Notably, the results emerging from hypertensive stratification revealed that plasma sortilin concentration was higher in hypertensive patients with uncontrolled BP than in controlled hypertensive patients and normotensive counterparts. Moreover, plasma levels of ASMase activity, S1P, and sNOX2-dp, key mediators involved in the deleterious effects of sortilin, followed the same modification pattern observed in sortilin circulating levels. In agreement with the results obtained in mice demonstrating how sortilin signaling alters sphingolipid metabolism, thereby promoting endothelial dysfunction and arterial hypertension, our data in humans suggest that sortilin and its mediators may represent new biomarkers for the prediction of vascular dysfunction and high BP. This hypothesis is also supported by the positive correlations found in our cohort of hypertensive patients between plasma levels of sortilin and S1P and between the latter and sNOX2-dp content, thus confirming the interdependency among sortilin, dysregulated sphingolipid metabolism, and oxidative stress in humans with arterial hypertension.

To the best of our knowledge, this is the first study to provide mechanistic insight into how sortilin orchestrates complex intracellular signaling to control vascular function and BP homeostasis, highlighting sortilin as a potential target in arterial hypertension. Moreover, our findings may add new aspects to the pathophysiology of the sphingolipid pathway, revealing the mechanism by which an alternative signaling pathway involving ASMase drives to a dysregulated sphingolipid signaling, leading to deregulation of cardiovascular function. Thus, although sphingolipids are essential regulators of cardiovascular homeostasis, our results could offer a plausible explanation for the altered sphingolipid levels observed in several cardiovascular diseases (44, 45). Few studies have reported the ability of some antihypertensive drugs, including angiotensin II receptor blockers (46, 47), calcium channel blockers, and beta blockers (48), to modulate pathways that integrate into S1P signaling, but to date, preclinical and clinical studies investigating the specific interaction between antihypertensives drugs and sphingolipid-related signaling pathways are still lacking. Even though our cohort of hypertensive patients was receiving antihypertensive treatment, we demonstrated that plasma sortilin concentrations were not influenced by drug therapy.

Despite the efficacy of current drug treatments, the difficulty in achieving the optimal BP levels by a large percentage of hypertensive patients reveals the importance of developing new preventive approaches. Although several GWAS studies have identified 120 loci associated with BP regulation, these explain only a tiny fraction of the phenotypic variants of BP, thus resulting in a considerable proportion of missing heritability (49, 50).

Additionally, hypertension is a highly heterogeneous disorder, and most individual biomarkers are only modestly associated with hypertension risk (51–54), which makes them impractical for clinical use. Owing to their intracellular signaling, several circulating molecules are mechanistically correlated with each other. Hence, monitoring multiple biomarkers and targeting the pathways in which they are involved could provide the chance to develop disease-modifying therapy and reduce the incidence of cardiovascular diseases. Although not recommended in current guidelines (49), increasing evidence strongly suggests the potential use for a biomarker-based strategy for the stratification and clinical management of hypertension (55).

In our study, the strong mechanism provided and the observation that sortilin, ASM, S1P, and sNOX2-dp level increases were more pronounced in uncontrolled hypertensive patients add new clinical perspectives for future development of multiple biomarker-based approaches, aiming at improving the ability to predict and monitor the progression of hypertension. A better understanding of the molecular mechanisms involved in the pathogenesis of hypertension will allow researchers to predict the lack of response to standard pharmacological treatment and prevent the onset of polypharmacy side effects that also compromise the patient’s health status and favor the development of chronic complex conditions.

It is important to point out a few limitations of our study. First, our study is based on a set of homogeneous groups of patients in terms of ethnicity and geography. Our selection criteria may not rule out the possible influence of other endothelial stressors that could be revealed in a population at higher risk of cardiovascular disease. Indeed, elevated circulating levels of sortilin were previously observed in statin‑naive subjects with coronary artery disease who underwent bypass surgery (7). Therefore, in this study, we selected patients with a prevalent hypertensive phenotype to pinpoint the role of sortilin in developing this pathophysiological condition in the absence of other influences. At this time, we can only speculate that the presence of more cardiovascular risk factors can further increase the circulating level of sortilin, which could be potentially used as an early biomarker for organ damage. Although our experimental studies provide strong evidence of the harmful effect of high circulating sortilin on endothelial function and BP regulation, the potential promise for sortilin assessment in clinical applications must await multicenter and prospective follow-up studies.

## Methods

### Reagents.

To characterize intracellular signaling, HUVECs or mesenteric arteries were pretreated with the following before data were obtained: 100 μM tempol (Sigma-Aldrich, 176141) for 1 hour; 1.5 μM K145 (Sigma-Aldrich, SML1003) for 1 hour; 5 μM (2R, 3S, 4E)-*N*-methyl-5-(4′-pentylphenyl)-2-aminopent-4-ene-1,3-diol (SK1-I) (Enzo, BML-EI411) for 1 hour; 100 nM W146 (Sigma-Aldrich, W1020) for 30 minutes; 5 μM *N*-(4-chlorophenyl)-3,3-dimethyl-2-oxobutanimidic acid, 2-(4-chlorophenyl)hydrazide (TY52156) (Cayman Chemical, 19119) for 30 minutes; 30 μM NSC23766 (Tocris, 2161) coincubated with sortilin for 1 hour; 1.5 μM ML171 (Tocris, 4653) for 30 minutes; GSK2795039 (MCE, HY-18950) for 30 minutes; 50 μM gp91 ds-tat (ANASPEC, AS-63818) for 30 minutes; and 200 U/mL PEG-SOD (Sigma-Aldrich, S9549) for 1 hour. Plasma sortilin was measured using an ELISA kit (Elabscience, E-EL-H5414). Acidic sphingomyelinase activity was determined in human plasma samples and cell lysates by the Acidic Sphingomyelinase Assay Kit (Abcam, ab190554).

### Mice.

C57BL/6J and homozygous *gp91^phox–/–^* mice (B6.129S-Cybb^tm1Din^/J, stock no. 002365) were obtained from Charles River Laboratories and The Jackson Laboratories, respectively. *S1P3*-knockout mice were generated by Jerold Chun (Sanford Burnham Prebys Medical Discovery Institute, La Jolla, California, USA). All experiments were conducted on 8-week-old male mice. We used C57BL/6J as WT and *gp91^phox–/–^* and *S1P3^–/–^* mice (both in a C57BL/6J background) on a regular diet. Animals were randomly divided into control and sortilin-treated groups and had free access to standard mouse chow and water. Mice were housed in groups (4–6 mice per cage) in a specific pathogen–free controlled environment under a normal 12-hour light/12-hour dark cycle.

### Cell culture.

HUVECs were obtained from the American Type Culture Collection (ATCC, PCS-100-010) and cultured in Vascular Cell Basal Medium (ATCC, PCS-100-030) supplemented with Endothelial Cell Growth Kit (ATCC, PCS-100-041) before experiments were performed. Cells were maintained at 37°C and 5% CO_2_ in a humidified incubator. Before experiments, HUVECs were seeded in 24-well plates or 60 mm or 100 mm dishes and grown until 80% confluency.

### Human subjects: Campania Salute Network.

Seventy-one patients with hypertension (defined as diastolic BP [DBP] ≥90 mmHg and systolic BP [SBP] ≥140 mmHg or on the basis of use of antihypertensive medication) and 71 healthy donor control subjects belonging to the Campania Salute Network Registry (https://www.campaniasalute.com/) were studied. Campania Salute is an open registry collecting information from a network of general practitioners and community hospitals networked with the Hypertension Center of Federico II University Hospital (Naples). The database generation of the Campania Salute Network was approved by the Federico II University Hospital Ethic Committee.

Other plasma samples from the Campania Salute Network Registry were collected from a small cohort of subjects who underwent ankle-brachial index (ABI) measurement and Doppler examination. A total of 36 healthy donor control subjects and 36 hypertensive patients with no diagnosis of PAD were included in the analysis. All patients had no history of previous cardiovascular diseases, symptomatic heart failure, ischemic heart disease, atrial fibrillation, stroke, or cognitive dysfunction.

### Moli-sani study.

289 plasma samples were collected from the biodata bank of the Moli-sani Study, a prospective cohort study established in 2005 to 2010 with an enrollment of 24,325 men and women (≥35 years of age) randomly recruited from the general population of Molise, a southern Mediterranean region in Italy (56). Nineteen hemolyzed samples were excluded from our analysis. A total of 270 patients were stratified into normotensive subjects (*n* = 89) and controlled (*n* = 91) or uncontrolled hypertensive subjects (*n* = 90), strictly matched and with no previous history of cardiovascular diseases. Hypertension was defined as controlled (SBP was <140 and DBP <90 mmHg with treatment) or uncontrolled (SBP was ≥140 and DBP ≥90 mmHg with treatment). All patients included in this study were not on statin therapy, which is known to influence circulating sortilin levels (57).

### Vascular reactivity studies.

Vascular reactivity studies were performed on second-order branches of the mesenteric artery, as previously described (58). Quantification of vasomotor response, BP, and molecular analyses were performed by a second individual who was blind to the genotype of the animal and/or the hypothesis that was being tested for each group. Vessels were isolated and dissected from fat and connective tissue in ice-cold Krebs solution and gassed with 95% O_2_ and 5% CO_2_. Subsequently, arteries were mounted on a pressure myograph in organ chambers with Krebs solution and treated with increasing concentrations of U46619 (10^–9^ to 10^–6^ M) in order to obtain a similar level of precontraction in each ring (80% of initial KCl-induced contraction). Caution was taken to avoid damage to the endothelium; functional integrity of the endothelium was confirmed by the vasodilation response to acetylcholine (10^–9^ to 10^–6^ M). Vasorelaxation was expressed as a percentage reduction of U46619-induced contraction.

### Gene silencing.

Second-order branches of the mesenteric arterial tree were removed from C57BL/6 mice and transfected with *PKC*ɛ siRNA (Santa Cruz Biotechnology Inc., sc-36250); *ASMase* siRNA (Santa Cruz Biotechnology Inc., sc-41651); *SphK1* siRNA (Santa Cruz Biotechnology Inc., sc-45446); *SphK2* siRNA (Santa Cruz Biotechnology Inc., sc-39226), or *aCDase* siRNA (Santa Cruz Biotechnology Inc., sc-140807) and their relative scramble vectors for 6 hours as previously described (59). Vessels were placed in a Mulvany pressure system filled with Krebs solution supplemented with 20 μg of siRNA vector. All vessels were perfused at 100 mmHg for 1 hour and then at 60 mmHg for 5 hours. Endothelium-dependent and independent relaxation were assessed by measuring the dilatory responses of mesenteric arteries to cumulative concentrations of acetylcholine (from 10^−9^ M to 10^−5^ M) or nitroglycerin (from 10^−9^ M to 10^−5^ M) in vessels precontracted with U46619 at a dose necessary to obtain a similar level of precontraction (80% of initial KCl-evoked contraction) in each vessel (60). Values are reported as a percentage of lumen diameter change after exposure to the substance. Responses were tested before and after transfection with described plasmids, in the presence or absence of sortilin (2.5 ng/mL for 1 hour).

### NO detection.

NO production in response to different stimuli as reported in figure legends was assessed and imaged using 4-amino-5-methylamino-2′,7-difluorofluorescein diacetate (DAF-FM) (Invitrogen, D-23842) as previously described (58). Isolated mesenteric arteries were embedded in a freezing medium to obtain transverse sections. Cryostat-frozen cross sections (10 μm) were incubated with DAF-FM (12.5 μM) diluted in phosphate buffer with 0.4 mM CaCl_2_ and incubated in a light-protected humidified chamber at 37°C for 1 hour. Slices were washed in PBS and then mounted on a glass slide for fluorescence microscopy. Images were acquired by fluorescence microscopy (Zeiss) and then analyzed using ImageJ software (developed by Wayne Rasband, NIH). The fluorescence intensity was calculated using integrated density (area × mean fluorescence) by measuring the mean optical fluorescence density.

To evaluate the nitrite level in the organ bath solution from the pressure myograph, we used 280i Nitric Oxide Analyzer (Sievers Instruments), as previously described (58). Briefly, prior to analysis, a mixture of glacial acetic acid and 1.0 mL of 0.5 M ascorbic acid was added to the purge bath to generate a calibration curve for nitrite. All samples were thawed in the dark just prior to analysis and kept on ice until injected. The concentration of nitrite in the samples was determined using the purge system of a Sievers Instruments model 280i nitric oxide analyzer. The specificity for both NO detection methods was confirmed by using the eNOS inhibitor N(ω)-nitro-l-arginine methyl ester (L-NAME) on mesenteric arteries (300 μmol/L for 30 minutes; Sigma-Aldrich, N5751).

### Measurement of ROS production by sensitive fluorescent indicator dihydroethidium.

Dihydroethidium (DHE) was used to evaluate the levels of oxidative stress in mesenteric arteries, HUVECs, and kidney cryosections. Vessels and kidney were immediately snap-frozen with OCT embedding compound in isopentane prechilled with liquid nitrogen, and transverse sections (7 or 10 μm) were produced using a cryostat (Leica CM1250). Sections were then incubated with 2 or 100 μmol/L DHE (Sigma-Aldrich, 309800) for 20 minutes at 37°C in a humidified chamber protected from light and then observed under a fluorescence microscope (Zeiss), as previously described (61). Fluorescence intensity was quantified as arbitrary units using ImageJ software.

To quantify O_2_^−^ in HUVECs, we measured DHE fluorescence using a microplate reader. At the end of the treatments, cells were incubated with a final DHE concentration of 5 μM for 30 minutes. Fluorescence was determined, in duplicate, using a microplate reader at excitation and emission wavelengths of 490 and 570 nm, respectively.

### Lipid peroxidation kit.

The levels of lipid peroxidation in mouse plasma samples and kidney homogenate were determined by quantifying the concentration of malondialdehyde (MDA) using a commercially available assay kit (Sigma-Aldrich, MAK085). Absorbance was measured using an Infinite Pro M200 Tecan microplate reader at 532 nm.

### Evaluation of NADPH-mediated O_2_^−^ production.

NOX-mediated superoxide radical (O_2_^–^) production was evaluated by using the lucigenin-enhanced chemiluminescence (ECL) assay, as previously described (62). After reaching confluence, HUVECs were washed with PBS, detached using 0.25% trypsin/EDTA (1 mmol/l), and resuspended in modified HEPES buffer containing 140 mmol/l NaCl, 5 mmol/l KCl, 0.8 mmol/l MgCl_2_, 1.8 mmol/l CaC_l2_, 1 mmol/l Na_2_HPO_4_, 25 mmol/l HEPES, and 1% glucose, pH 7. Subsequently, cells were homogenized, and a total of 100 μg of extract was distributed on a 96-well microplate. Vessels were incubated in Krebs buffer and equilibrated for 30 minutes at 37°C.

Vessels were homogenized in a buffer containing protease inhibitors (20 mmol/L monobasic potassium phosphate, 1 mmol/L EGTA, 0.01 mmol/L aprotinin, 0.01 mmol/L leupeptin, 0.01 mmol/L pepstatin, 0.5 mmol/L phenylmethylsulfonyl fluoride, pH 7.0). Protein content was measured by the Bradford method. The reaction was started by the addition of NADPH (0.1 mmol/l) and lucigenin (5 μmol/l) to each well. Chemiluminescence was measured using Tecan Infinite Pro M200 multimode microplate at 37°C.

### Flow cytometry.

HUVEC single-cell suspensions were stained with mouse anti-human S1P3/EDG-3 PE-conjugated antibody (Monoclonal Mouse IgG2A, clone 776808, R&D Systems). After 30 minutes incubation at 4°C in the dark, cells were washed and resuspended in staining buffer (PBS 2% FBS) for flow cytometry acquisition and analysis using FACSVerse Flow Cytometer (BD Biosciences).

### RT-qPCR analysis.

Total RNA was extracted with TRI reagent (Sigma-Aldrich), quantified by NanoDrop 1000 spectrophotometer (Thermo Fisher Scientific), treated with DNase I (Invitrogen), and reverse transcribed using a SuperScript VILO Master Mix (Invitrogen, 11755500). Specific cDNAs were amplified and analyzed using a 7500 Real-Time PCR System (Applied Biosciences). Details of PCR primers for *S1P3* (63), *NOX2*, *NOX4*, and *GAPDH* are provided in Supplemental Table 3. Quantification was performed using ΔCT calculation.

### Immunofluorescence and confocal fluorescence imaging.

Confluent HUVECs seeded onto 12 mm glass coverslips were pretreated with TY-52156 (30 minutes) inhibitor or vehicle (DMSO 0.02%) prior to sortilin and fixed with 4% paraformaldehyde in 0.1 M PBS buffer for 15 minutes at room temperature. Cells were permeabilized using PBS containing 0.005% saponin for 30 minutes and incubated overnight at 4°C with IgG primary rabbit polyclonal anti-rac1 (Abcam, anti-Rac1 antibody catalog ab97732), or anti–VE-cadherin (Abcam, catalog ab33168), followed by a 1-hour incubation with the secondary antibodies Alexa Fluor 488–conjugated anti-rabbit IgG (Invitrogen, catalog A11008) or DyLight 549–conjugated anti-rabbit IgG (Vector Laboratories, catalog DI-1549).

For immunofluorescence, frozen mesenteric artery sections (7 μm) were fixed in ice-cold acetone for 5 minutes at –20°C, air-dried, blocked (3% BSA and 5% donkey serum in PBS), and then incubated overnight at 4°C with one of the following primary antibodies: mouse monoclonal anti-CD68 antibody (Abcam, catalog ab955,clone KP1), rat monoclonal anti-monocyte plus macrophage MOMA-2 antibody (Abcam, catalog ab33451, clone MOMA-2), rabbit polyclonal anti–3-nitrotyrosine antibody (Millipore, catalog 06-284), and sheep polyclonal anti–vWF antibody (Abcam, catalog ab11713), the latter used as a VE marker. The arteries were washed several times with PBS and incubated again for 1 hour with the following secondary antibodies: DyLight 549–conjugated anti-mouse IgG (Vector Laboratories, catalog DI-2549), Alexa Fluor 555–conjugated anti-rat IgG (Southern Biotec, catalog 6430-32), DyLight 549–conjugated anti-rabbit IgG (Vector Laboratories, catalog DI-1549), and FITC-conjugated anti-sheep IgG (Thermo Fisher Scientific, catalog A16049). Images were acquired using a confocal laser-scanning fluorescence microscope TCS SP5 (Leica Microsystems). Nuclei were stained with DAPI.

### Evans blue dye.

The functional integrity of the endothelial layer of mesenteric arteries was also assessed 14 days after treatment with vehicle or sortilin using Evans blue dye (Sigma-Aldrich), as previously described (64). Briefly, 2% Evans blue dye solution diluted in saline was injected into the tail vein 30 minutes before euthanasia, followed by PBS perfusion. Images were captured with an LSM 510 microscope (Carl Zeiss MicroImaging).

### Western blot.

For total protein extraction, pooled mesenteric arteries or cells were lysed in a buffer containing 150 mmol/L NaCl, 50 mmol/L Tris-HCl (pH 8.5), 2 mmol/L EDTA, 1% v/v NP-40, 0.5 % w/v deoxycholate, 10 mmol/L NaF, 10 mM sodium pyrophosphate, 2 mmol/L PMSF, 2 g/ml leupeptin, and 2 g/ml aprotinin (pH 7.4). Lysates were then centrifuged at 38,000*g* for 30 minutes at 4°C to collect the supernatant. Protein concentration was measured using a dye-binding protein assay kit (Bio-Rad) and read with the spectrophotometer at a wavelength of 595 nm. Immunoblotting was performed as previously described (65), using the following antibodies: anti–phospho-eNOS serine 1177 (Enzo Life Sciences, catalog ALX-804-396-C100, clone 15E2); anti-phospho-eNOS-Thr494 (Cell Signaling Technology, catalog 9574); anti-eNOS (Cell Signaling Technology, catalog 9570); anti–β-actin (Abcam, mAb, catalog ab8226, clone mAbcam 8226); anti–phospho-protein-tyrosine kinase 2-β phospho-Tyr579 (PYK2B pY579) (Elabscience, catalog E-AB-21240); anti–phospho-PKCɛ Ser729 (Biorbyt, catalog orb315664); anti–PKCα (phospho T497) antibody (Abcam, catalog ab76016, clone EP2608Y); anti–PKC-PAN (Sigma-Aldrich, catalog SAB4502356); anti-ASMase (SMPD1; Biorbyt, catalog ORB214591); anti-S1P1 (Immunological Sciences, catalog AB-83739); anti-S1P3 (Elabscience, catalog E-AB-31267); anti-gp91phox (Santa Cruz Biotechnology Inc., catalog sc-130543, clone 54.1); anti-SphK1 (Santa Cruz Biotechnology Inc., catalog sc-365401, clone G-11); anti-SphK2 (MyBioSource, catalog MBS2518663); anti-aCDase (ASAH1; MyBioSource, catalog MBS1492517), and anti-active Rac1 (Rac1-GTP) (Neweast Bioscience, catalog 26903). Secondary antibodies were purchased from Amersham Life Sciences (GE Healthcare). Bands were visualized with ECL (Amersham Life Sciences) according to the manufacturer’s instructions. Immunoblotting data were analyzed using ImageJ software to determine density of the bands. See complete unedited blots in the supplemental material.

### Measurement of sphingolipids in cells.

Confluent HUVECs (ATCC, PCS-100-010) were incubated with recombinant sortilin (2.5 ng/mL, 1 hour) diluted in Vascular Cell Basal Medium (ATCC, PCS-100-030) and supplemented with Endothelial Cell Growth Kit (ATCC, PCS-100-041). The culture medium and cell lysates were analyzed for extracellular S1P and intracellular ceramide content, respectively.

### Sphingolipid analysis by LC-MS/MS.

All solvents and additives were LCMS grade and purchased from Sigma-Aldrich. Internal standard of sphingosine-1-phosphate, ceramides C16, C18, and C24:1, and deuterated ceramide mix were purchased from Avanti Polar Lipids (Alabaster). For the extraction of sphingolipids, human plasma (20 μl) was diluted to 400 μL with Milli-Q water (Sigma-Aldrich), and 3 μl of the internal standard was added, after which samples were extracted by the addition of 400 μL of isopropanol/ethyl acetate 18/85 (v/v); the solution was vortexed for 20 seconds and centrifuged at 16,110 x *g* for 5 minutes. The upper organic phase was collected, while the aqueous phase was acidified with 20 μL of formic acid and reextracted with 400 μL of fresh isopropanol/ethyl acetate 18/85 (v/v%). The obtained solution was vortexed and centrifuged. The supernatants were pooled and dried under a gentle stream of nitrogen. For sphingolipid extraction, cell media (500 μL) was diluted with 1 mL isopropanol/ethyl acetate 18/85 (v/v%) and processed as described above. Samples were resolubilized with 150 μL of methanol and 1 mM HCOONH_4_ plus 0.2% HCOOH (v/v). UHPLC-MS/MS analysis was carried out with a Shimadzu Nexera coupled online to a triple-quadrupole LCMS 8050 (Shimadzu) by an ESI source.

### UHPLC-MS/MS conditions.

Separation was performed on a Kinetex EVO C18, 150 mm × 2.1 mm, 2.6 μm (Phenomenex), at a flow rate of 0.5 mL/min, employing as mobile phase (a) ACN/H_2_O: 60/40 10 mM HCOONH_4_ plus 0.1% HCOOH (v/v%) and (b) isopropanol/ACN 90/10 plus 0.1% HCOOH (v/v%) with the following gradient: 0 minutes, 5% b; 0 to 2.50 minutes, 50% b; 2.51 to 7 minutes, 50% to 99% b; isocratic for 2.50 minutes; returning to 5% in 4 minutes. 5 μL were injected. The ESI was operated in positive ionization. MS source parameters were as follows: interface temperature, 300°C; desolvation line temperature, 250°C; and heat block temperature, 400°C. Nebulizing gas, drying gas, and heating gas were set to 3, 10, and 10 L/min, respectively. MS/MS analysis of sphingolipids was performed in multiple reaction monitoring (MRM). The SRM transition of nonavailable ceramides was optimized in silico using the Lipid Creator tool in the freely available Skyline open source (66).

### In vivo sortilin administration.

To assess the acute effect of sortilin on BP control, mice received a single i.p. injection of sortilin (40 ng/mL, i.p.), and BP was measured every hour until 6 hours after injection. In another set of experiments, WT mice were injected with TY52156 (S1P3 antagonist, 3 mg/kg i.p.) or vehicle (DMSO, 0.05%) 2 hours after sortilin administration and BP was measured at the same time points. For chronic sortilin infusion, mice were treated with sortilin (40 ng/mL/d) or vehicle alone (saline solution 0.9%) via s.c. osmotic minipumps (ALZet 1002). For osmotic pump implantation, mice were anesthetized with 1.5% to 2% isoflurane. A 1 cm incision was made in the right dorsum. Then the pump containing recombinant sortilin protein or vehicle (saline solution, 0.9%) was inserted in 8-week-old WT, *S1P3^–/–^*, and *gp91^phox–/–^* male mice and the wound was closed with sutures. After implantation, mice were injected with carprofen (5 mg/kg, s.c.) as a postoperative analgesic. BP was measured every other day from day 0 to day 14 of vehicle or sortilin infusion. At the end of treatment, animals were sacrificed under terminal isoflurane anesthesia.

### BP measurements in mice.

BP was measured in conscious mice by the tail-cuff system (BP-2000, VisiTech Systems), as previously described (65), and by the telemetry system (DSI HD-X11, Data Science International). Briefly, animals were placed in a plastic chamber maintained at 34°C, and a cuff with a pneumatic pulse sensor was attached to the tail. For the tail-cuff system, mice were subjected to at least 7 days of training, and basal BP levels were recorded as the average of values determined on at least 3 days. For the telemetry method, BP measurement was performed on the basis of a protocol provided by Data Science International. Mice were anesthetized with isoflurane. After the left common carotid artery was isolated, the catheter that was connected to the transducer was introduced into the carotid and advanced toward the aortic arch. The transducer was implanted into the abdomen s.c. All procedures were completed under sterile conditions. Transmitters were detected by receiver platforms placed directly underneath the animals’ cages. Mice were allowed to recover for 1 week, and BP and heart rate before and during sortilin treatment were recorded continuously in freely moving animals. Data were recorded daily for 2 hours (10 am to 12 pm).

### Evaluation of human endothelial function by endoPAT.

Endothelial function was evaluated in patients from the Campania Salute Network project using the EndoPAT 2000 device (Itamar Medical Ltd.) for the determination of RHI, as previously described (29). Briefly, measurements were performed according to the manufacturer’s instructions and were calculated using a computerized automated algorithm (version 3.1.2) provided with the device. Brieﬂy, subjects were in a supine position for a minimum of 15 minutes before measurements in a quiet, temperature-controlled (21–24°C) room with dimmed lights. The subjects were asked to remain as still as possible and silent during the entire measurement period. Each recording consisted of 5 minutes of baseline measurement, 5 minutes of occlusion measurement, and 5 minutes of postocclusion measurement (hyperemic period). Occlusion of the brachial artery was performed on the nondominant, upper arm. The occlusion pressure was at least 60 mmHg above the SBP (minimally 200 mmHg and maximally 300 mmHg).

### Vascular examination for assessment of PAD.

ABI measurement and Doppler waveform analysis of ankle arteries were determined according to current guidelines (67). An ABI greater than or equal to 0.9 or less than 1.3 was considered normal. Hypertensive patients with clinical findings consistent with PAD were excluded from the analysis.

### sNOX2-dp ELISA detection.

NOX2 activation was measured in human plasma as sNOX2-dp with an ELISA method as previously reported (68). Briefly, the peptide was recognized by binding to a specific monoclonal antibody against the amino acidic sequence (aa 224–268) of the extra membrane portion of NOX2, which was released following platelet activation.

Enzyme activity was measured spectrophotometrically by increased absorbency at 450 nm. Since the increase in absorbency is directly proportional to the amount of sNOX2-dp of the unknown sample, the latter can be derived by interpolation from a reference curve generated in the same assay with reference standards of known concentrations of sNOX2-dp (0–200 pg/ml). Values were expressed as pg/ml; intraassay and interassay coefficients of variation were 8.95% and 9.01%, respectively.

### Statistics.

Data are represented as mean ± SD. The Shapiro-Wilk or Kolmogorov-Smirnov tests were used to evaluate the normality of distribution of investigated parameters. Two-sided unpaired Student’s *t* test was performed for comparisons between 2 independent groups. One-way ANOVA (with Bonferroni’s correction) or, when normality tests failed, nonparametric Kruskal-Wallis test (with Dunn’s multiple comparison) was used for comparisons of multiple means. Two-way ANOVA (with Bonferroni’s correction) was used for comparisons of concentration-response curves. Two-way repeated measurements (RM) ANOVA (with Bonferroni’s correction for comparison of multiple means) was used to calculate statistical significance of BP measurements in mice. Fisher’s exact test or, where appropriate, χ^2^ test was used for comparison of categorical data between study subjects. Pearson’s correlation analysis was used to measure the degree of correlation between 2 variables. The gradient of linear model and its 95% CI have been calculated to verify whether there was a statistically significant difference between the 2 variables. A *P* value of less than 0.05 was considered significant. To verify whether there was an effect of antihypertensive drugs on sortilin plasma levels, N-way factorial ANOVA was performed with a significance level of 0.05. Statistical analysis was performed with GraphPad Prism software (version 8.0, GraphPad Software), and MATLAB software (version R2019b, The Mathwork Inc.). Effect sizes and their confidence intervals were calculated with the MATLAB-based Measures-of-Effect-Size-toolbox (69).

### Study approval.

Mouse studies were carried out in accordance with the *Guide for the Care and Use of Laboratory Animals* (National Academies Press, 2011) and were approved by the IRCCS INM Neuromed review board (no. 1070/2015 PR). All human data were collected in accordance with the Declaration of Helsinki and with local Ethics Committee (University of Naples, Naples Italy and Catholic University of Rome, Rome, Italy) approval. We included individuals from two prospective Italian studies, The Campania Salute Network Study (University of Naples Federico II, Naples, Italy; permit number 16/14) and the Moli-sani Study (Catholic University of Rome, Rome, Italy; permit number P99, A.931/03-138-04/C.E./2004). Written, informed consent was obtained from all included participants.

## Author contributions

PDP, AC, and CV designed studies and wrote the manuscript. ES and FM performed LC-MS/MS analysis. PDP, MO, and EC performed cell-culture studies. LI, ADC, RI, VT, NV, AS, and MC provided human samples. GG performed screening tests for PAD. AD, MDL, and EV performed mouse studies. RC, VC, and PI performed molecular studies. FA performed statistical analysis. SM analyzed data. BL provided S1P3-knockout mice. VI, LI, PC, BL, AAP, and CV reviewed and edited the manuscript.

## Supplementary Material

Supplemental data

## Figures and Tables

**Table 1:**
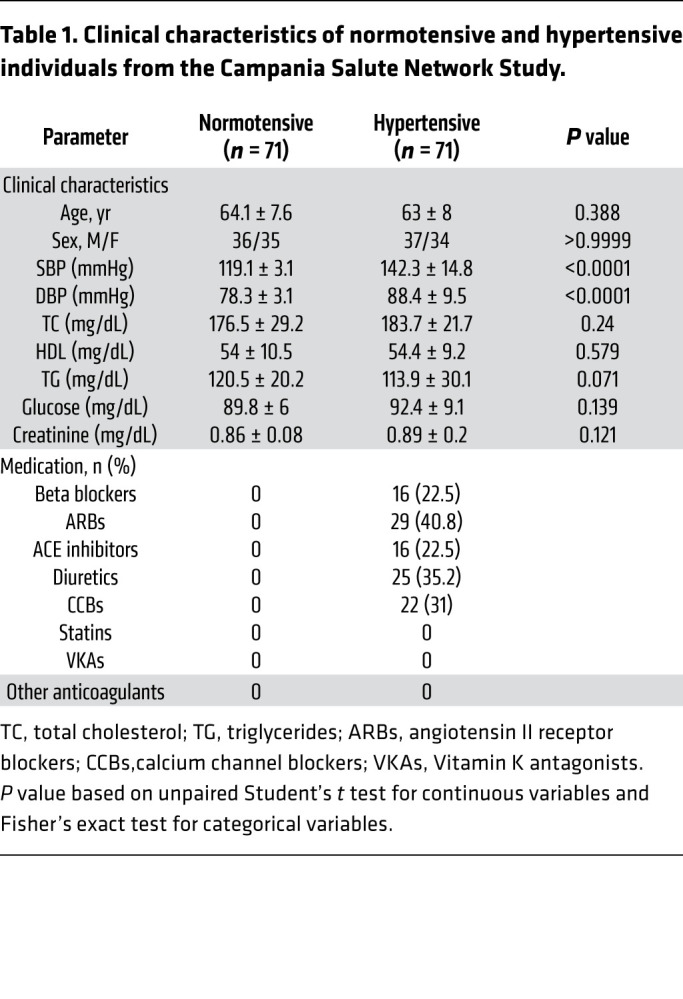
Clinical characteristics of normotensive and hypertensive individuals from the Campania Salute Network Study.

**Table 2:**
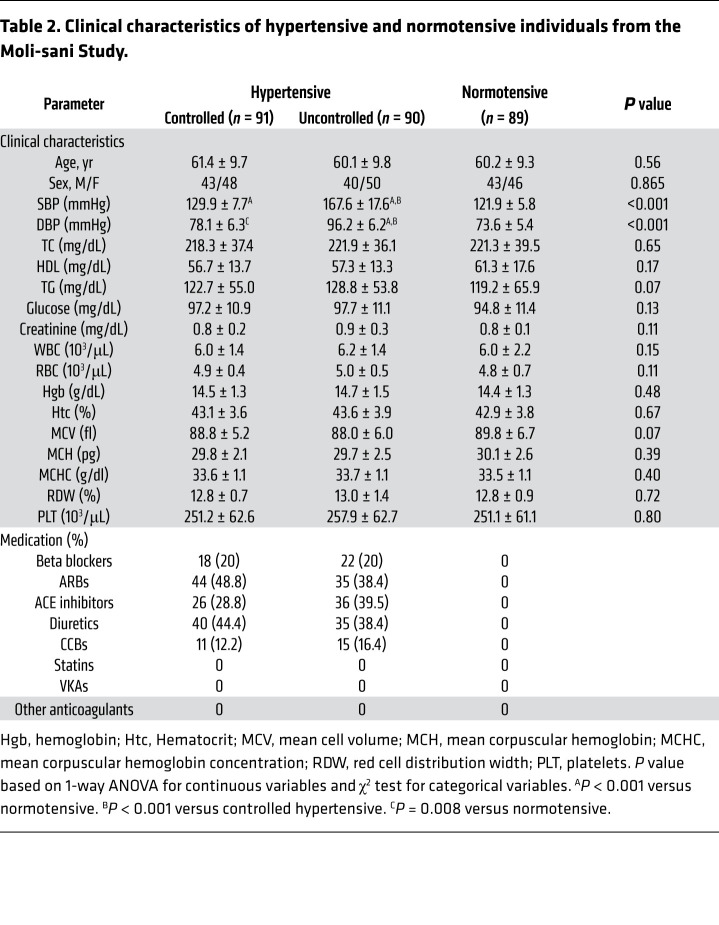
Clinical characteristics of hypertensive and normotensive individuals from the Moli-sani Study.

## Version 1 02/01/2022

Electronic publication
