# Supplementary material for: Targeting the ASMase/S1P pathway protects from sortilin-evoked vascular damage in hypertension
Source: J Clin Invest. 2022 Feb 1;132(3):e146343. doi: 10.1172/JCI146343 (PMC8803332; doi:10.1172/JCI146343)
Supplement: Supplemental data [file jci-132-146343-s115.pdf]

SUPPLEMENTAL DATA

Targeting ASMase/S1P pathway protects from Sortilin-evoked vascular damage in hypertension

Paola Di Pietro, Albino Carrizzo, Eduardo Sommella, Marco Oliveti, Licia Iacoviello, Augusto Di Castelnuovo, Fausto Acernese, Antonio Damato, Massimiliano De Lucia, Fabrizio Merciai, Paola Iesu, Eleonora Venturini, Raffaele Izzo, Valentina Trimarco, Michele Ciccarelli, Giuseppe Giugliano, Roberto Carnevale, Vittoria Cammisotto, Serena Migliarino, Nicola Virtuoso, Andrea Strianese, Viviana Izzo, Pietro Campiglia, Elena Ciaglia, Bodo Levkau, Annibale A. Puca, and Carmine Vecchione

Supplemental Figure 1

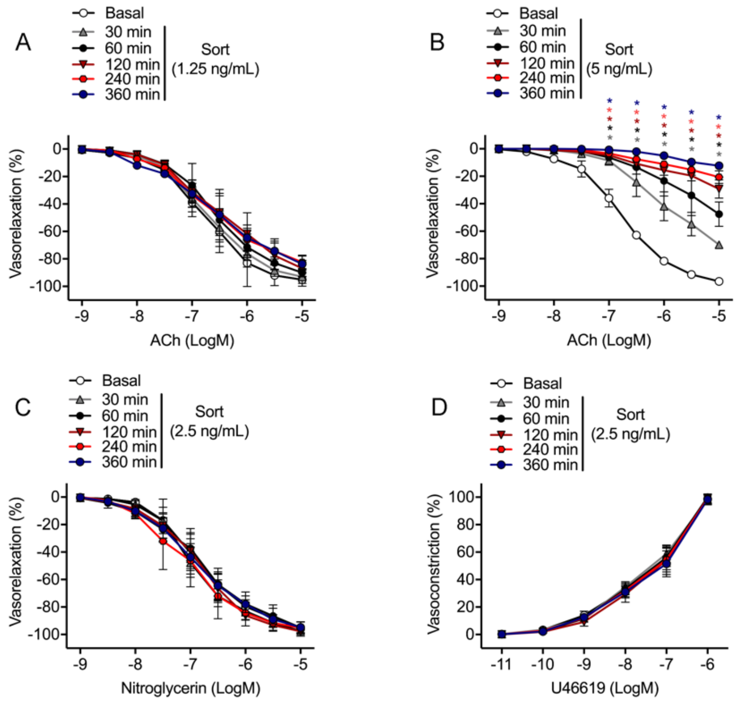

Supplemental Figure 1 Sortilin does not affect endothelial-independent vasorelaxation and vasoconstriction. (**A**) Acetylcholine (ACh)-evoked vasorelaxation in WT mice mesenteric arteries exposed to vehicle or sortilin (sort) at 1.25 ng/mL or to (**B**) 5 ng/mL for different preincubation times (30, 60, 120, 240, and 360 minutes); (n=3-6). Concentration-response curves to (**C**) nitroglycerin (*n*=3-4) and (**D**) to thromboxane mimetic U46619 (*n*=6) in WT mesenteric arteries treated with vehicle or 2.5 ng/mL of sortilin at different incubation times. Results are expressed as mean ± SD. Statistical analysis was performed by two-way ANOVA followed by Bonferroni's *post hoc* test. (**B**) * $P<0.0001$ vs. Basal at the same ACh concentration (as indicated by color code).

Supplemental Figure 2

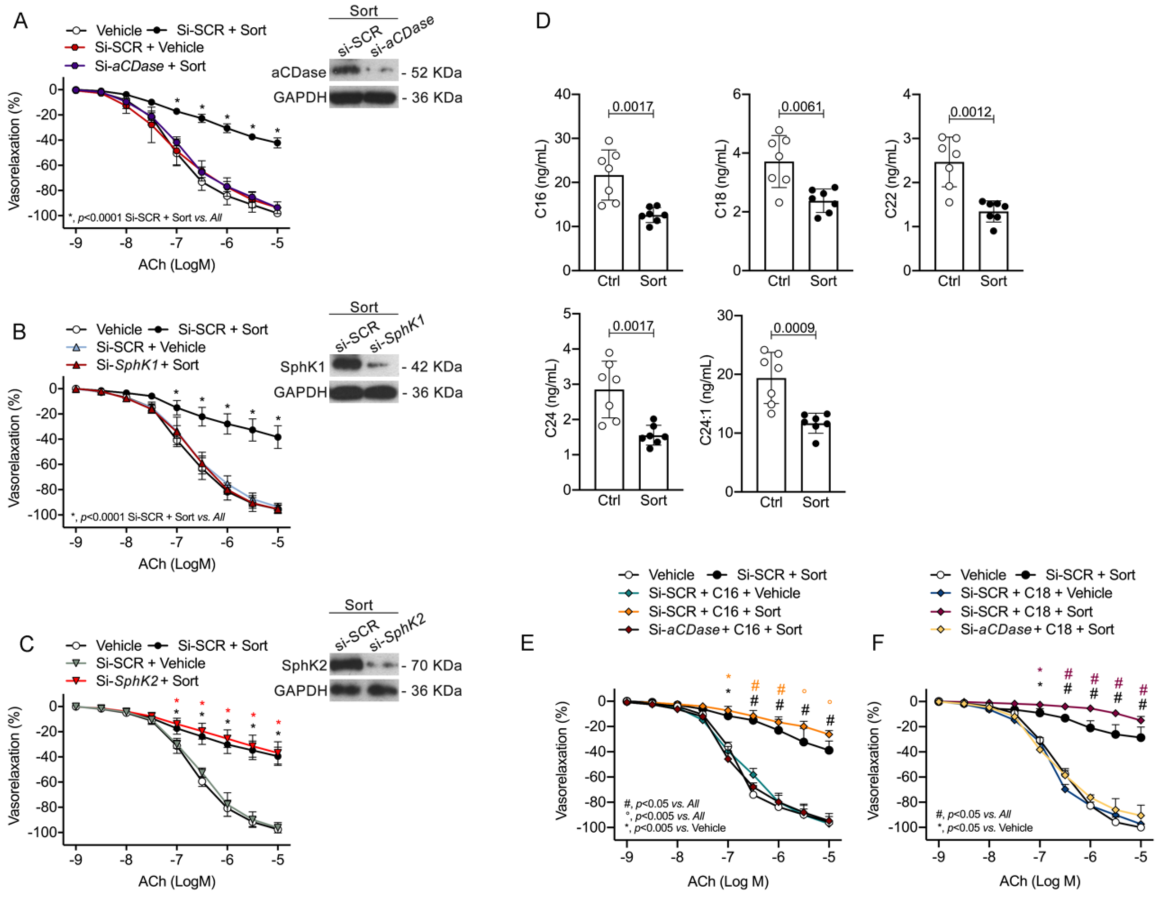

Supplemental Figure 2 Sortilin induces endothelial dysfunction by altering sphingolipid metabolism. (**A**) ACh-evoked vasorelaxation in WT mesenteric arteries exposed to vehicle or pretransfected with either siRNA targeting *aCDase* or a scrambled siRNA (si-SCR) and then exposed to vehicle or sortilin for 60 minutes. The effectiveness of silencing was determined by western blotting; (*n*=5). (**B** and **C**) ACh-evoked vasorelaxation in WT mesenteric arteries exposed to vehicle or pretransfected with either *SphK1* siRNA, *SphK2* siRNA or a scrambled siRNA (si-SCR) prior to 1 h treatment with vehicle or sortilin. The effectiveness of silencing was determined by Western blotting; (*n*=4-5). (**D**) Individual sphingolipid species measured by LC-MS/MS in HUVECs treated with vehicle (ctrl) or sortilin for 1 hr; (*n*=7). (**E** and **F**) ACh-evoked vasorelaxation in WT mesenteric arteries exposed to vehicle or pretransfected with either siRNA targeting *aCDase* or si-SCR and then exposed to (E) C16 or (F) C18 prior to vehicle or sortilin for 60 minutes; (*n*=3). Results are expressed as mean ± SD. (**A-C, E** and **F**) Two-way ANOVA followed by Bonferroni's *post hoc* test or (**D**) unpaired two-tailed Student's *t*-test was used. (**C**) * $P<0.0001$ vs. Vehicle or Si-SCR + Vehicle at the same ACh concentration (as indicated by color code). (**E**) * $P<0.005$ vs. Vehicle; # $p<0.05$ vs. Vehicle, Si-SCR + C16 + Vehicle and Si-*aCDase* + C16 + Sort; ° $p<0.005$ vs. Vehicle, Si-SCR + C16 + Vehicle and Si-*aCDase* + C16 + Sort at the same ACh concentration (as indicated by color code). (**F**) * $P<0.05$ vs. Vehicle; # $p<0.05$ vs. Vehicle, Si-SCR + C18 + Vehicle and Si-*aCDase* + C18 + Sort at the same ACh concentration (as indicated by color code).

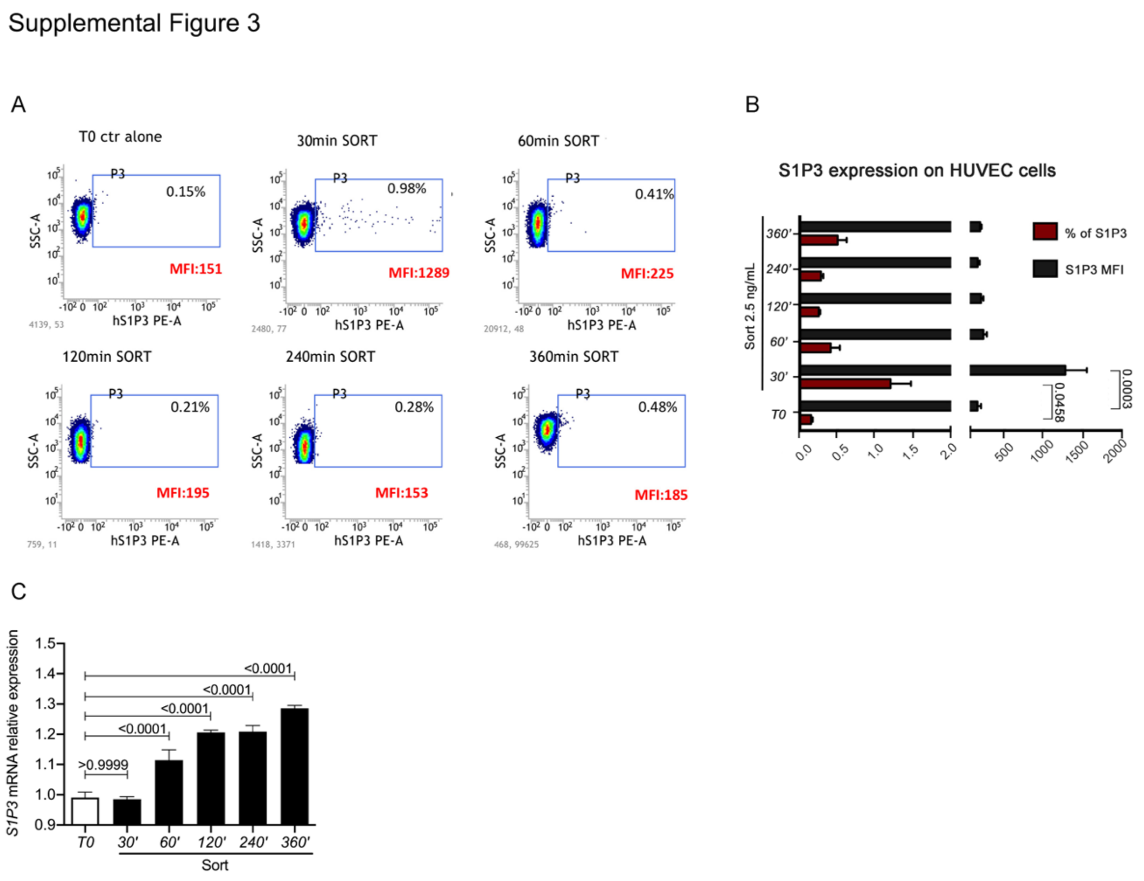

Supplemental Figure 3 S1P3 expression at different time points following sortilin stimulation in HUVEC cells. (**A**) Representative flow cytometry SSC vs S1P3 density plots for each experimental condition is presented. (**B**) Bars graph report both the percentage (*red*) of S1P3+ and mean fluorescence intensity (MFI) (*black*) of S1P3 receptor on viable HUVEC gated cells from 3 independent experiments. (**C**) mRNA expression of *S1P3* determined by quantitative reverse transcription polymerase chain reaction in HUVECs treated at different timepoints with 2.5 ng/mL of sortilin; (*n*=3). Results are expressed as mean ± SD. Statistical analysis was performed by one-way ANOVA followed by Bonferroni's *post hoc* test.

Supplemental Figure 4

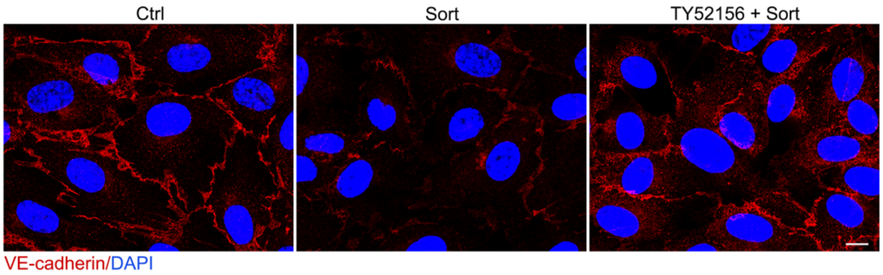

Supplemental Figure 4 Sortilin disengages VE-cadherin in HUVECs. Representative images of immunofluorescence staining of clustered VE-cadherin molecules in HUVECs treated with vehicle (ctrl), sortilin alone, or pretreated with the S1P3 inhibitor TY52156. DAPI-stained nuclei are in blue. Scale bar: 25 μm.

Supplemental Figure 5

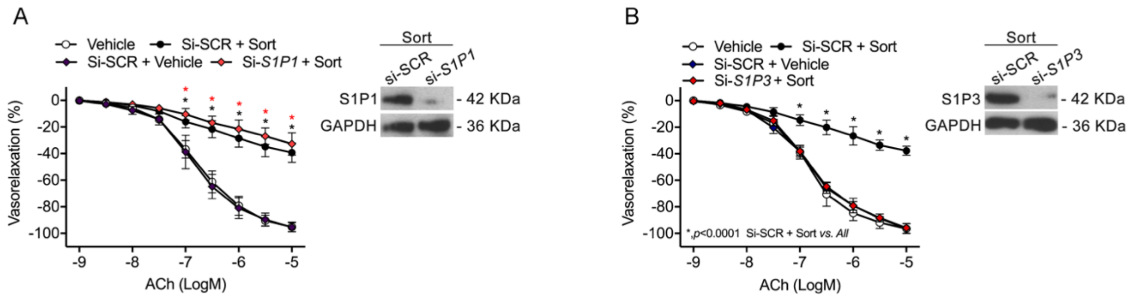

Supplemental Figure 5 Effects of *S1P1* or *S1P3* receptors silencing on sortilin-induced endothelial dysfunction. (**A** and **B**) ACh-evoked vasorelaxation in preconstricted WT mesenteric arteries exposed to vehicle or pretransfected with either *S1P1* siRNA, *S1P3* siRNA or a scrambled siRNA (si-SCR) prior to 1 hour treatment with vehicle or sortilin. The effectiveness of silencing was determined by Western blotting. GAPDH was used as a loading control; (*n*=3). Results are expressed as mean ± SD. Statistical analysis was performed by two-way ANOVA followed by Bonferroni's *post hoc* test. (**A**) * $P<0.0001$ vs. Vehicle or Si-SCR + Vehicle at the same ACh concentration (as indicated by color code).

Supplemental Figure 6

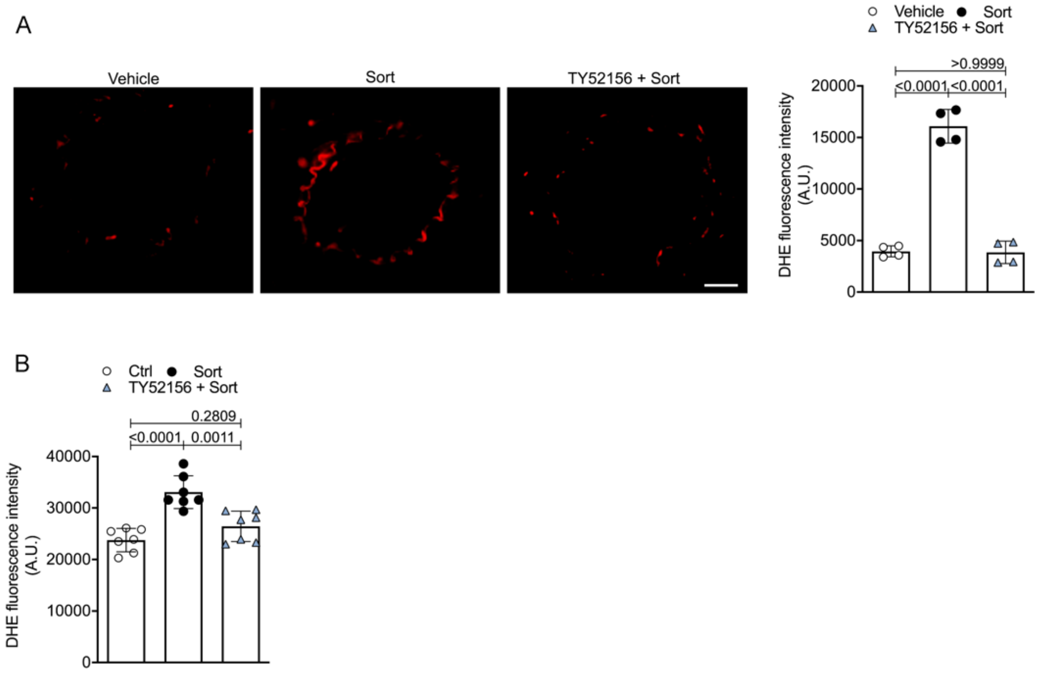

Supplemental Figure 6 TY52156 prevents sortilin-induced ROS overproduction. (**A**) Representative images of Dihydroethidium (DHE, 2 μmol/L) staining of WT mesenteric arteries treated with vehicle, sortilin alone, or pretreated with the S1P3 inhibitor TY52156. Scale bar: 25 μm. Bar graph shows DHE fluorescence intensity; (*n*=4). (**B**) DHE fluorescence measurement by microplate reader in HUVECs treated with vehicle, sortilin alone, or pretreated with the S1P3 inhibitor TY52156; (*n*=7). Results are expressed as mean ± SD. Statistical analysis was performed by one-way ANOVA followed by Bonferroni's *post hoc* test.

Supplemental Figure 7

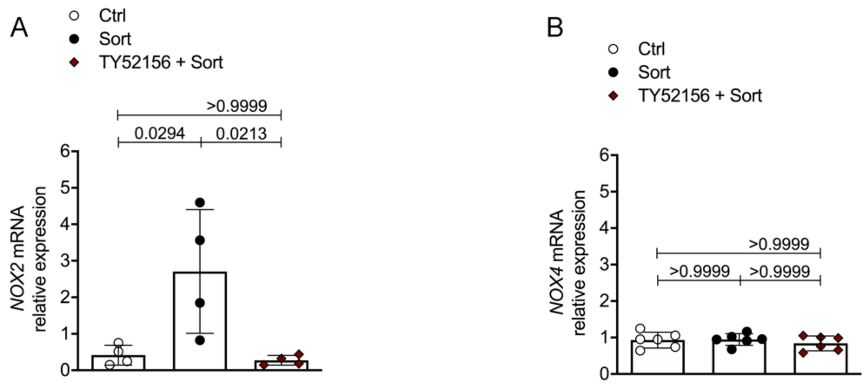

Supplemental Figure 7 Effect of sortilin on mRNA expression of NADPH oxidases isoforms in HUVECs. (**A** and **B**) mRNA expression of (**A**) *NOX2* and (**B**) *NOX4* determined by quantitative reverse transcription polymerase chain reaction in HUVECs treated with vehicle, sortilin alone, or pretreated with the S1P3 inhibitor TY52156; (*n*=4-6). Results are expressed as mean ± SD. Statistical analysis was performed by unpaired two-tailed Student's *t*-test.

Supplemental Figure 8

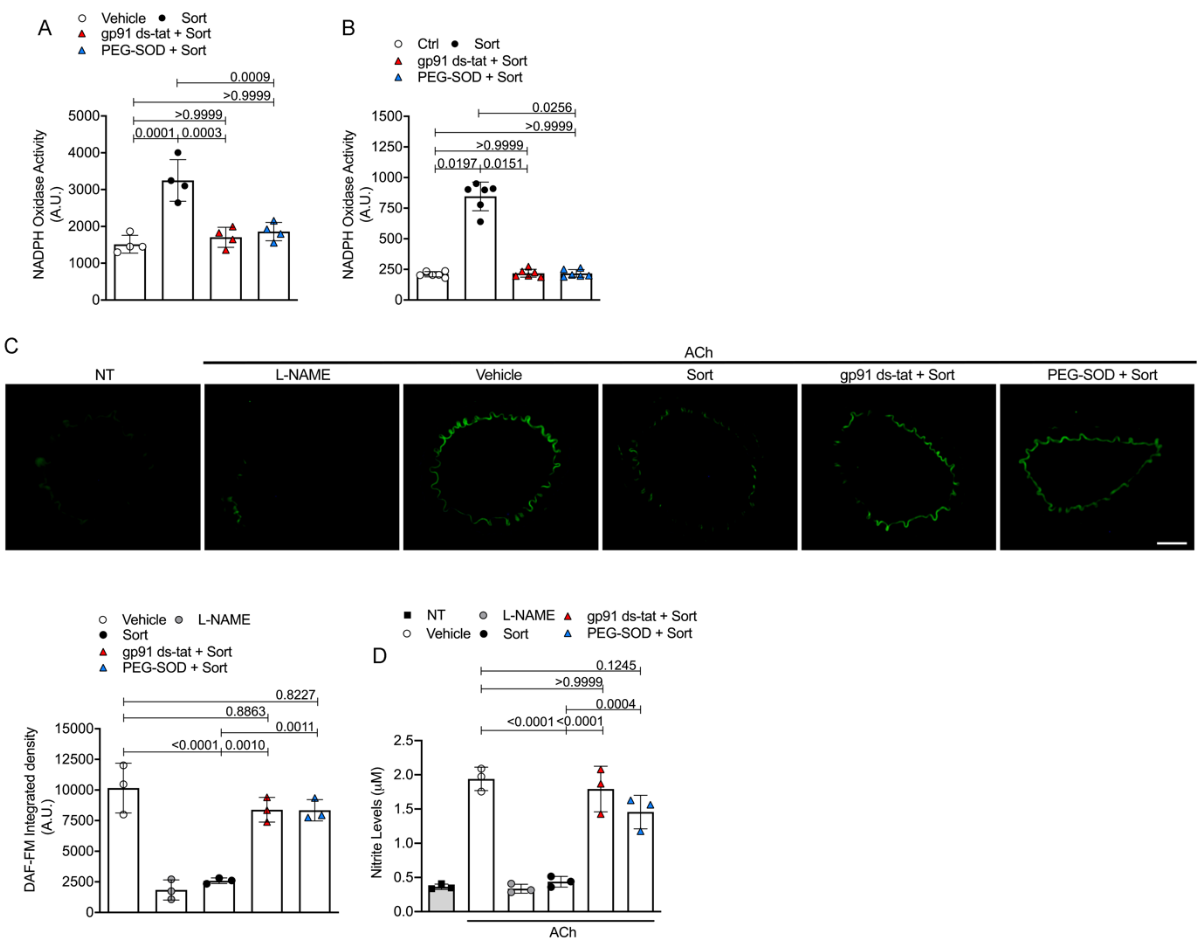

Supplemental Figure 8 NOX2 inhibition protects from oxidative stress and NO impairment induced by sortilin. (**A** and **B**) Effect of sortilin on NADPH oxidase activity in (A) WT mice mesenteric arteries and in (B) HUVECs in presence of gp91 ds-tat or PEG-SOD; (*n*=4; *n*=6, respectively). (**C**) Detection of NO by DAF-FM fluorescence in untreated WT mesenteric arteries (NT), stimulated with ACh alone (10^{-5} M) (vehicle) or in presence of L-NAME, sortilin alone or pretreated with either gp91 ds-tat or PEG-SOD prior to sortilin. Scale bar: 25 µm; bar graph gives mean of fluorescence integrated density of DAF-FM; (*n*=3). (**D**) NO metabolites concentration in supernatants of untreated mesenteric arteries (NT), stimulated with ACh alone (10^{-5} M) (vehicle) or in presence of L-NAME, sortilin alone or pretreated with either gp91 ds-tat or PEG-SOD prior to sortilin; (*n*=3). Results are expressed as mean ± SD. (**A, C** and **D**) One-way ANOVA followed by Bonferroni's *post hoc* test was used. (**B**) Non-parametric Kruskal–Wallis test with Dunn's correction was used.

Supplemental Figure 9

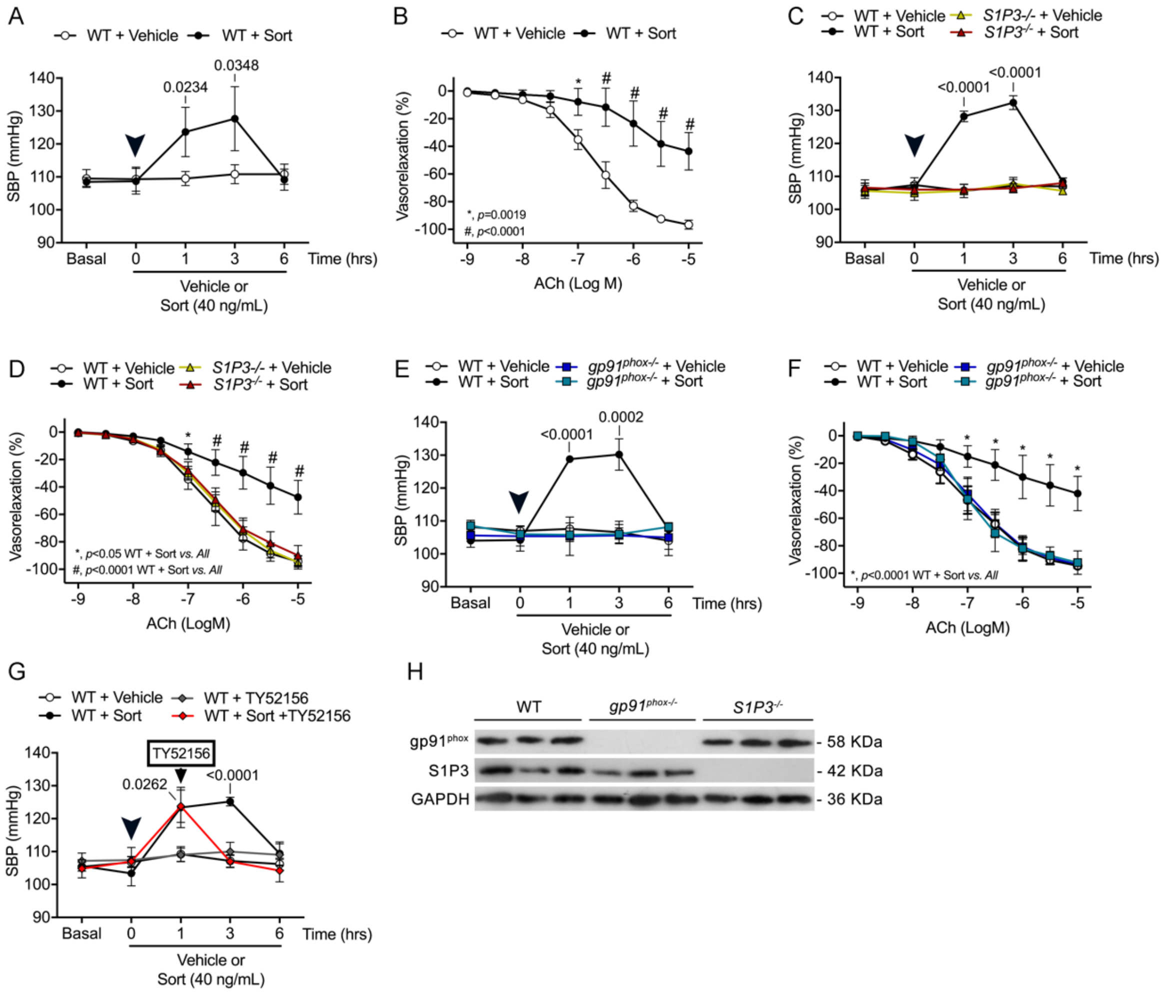

Supplemental Figure 9 Deletion of either *S1P3* or *gp91^{phox}* protects from hypertension and endothelial dysfunction induced by acute sortilin administration. (**A-F**) WT, *S1P3$^{-/-}$*, and *gp91$^{phox-/-}$* mice analyzed for (**A, C** and **E**) systolic blood pressure (SBP) before and after an intraperitoneal (i.p.) bolus dose of sortilin (40ng/mL); (*n*=5-6), and (**B, D** and **F**) for ACh-induced endothelium-dependent vasodilation in mesenteric arteries 6 hours after sortilin injection; (*n*=3-4 WT, *n*=5 *S1P3$^{-/-}$*, *n*=4 *gp91$^{phox-/-}$*). (**G**) Time course of SBP measured in WT mice before and after (arrowheads) a single dose of sortilin (40 ng/mL, i.p.). 2 hours after sortilin injection, mice were administered TY52156 (i.p.), and their blood pressure measured 2 and 4 hours later; (*n*=5). (**H**) Western Blot analysis conducted on extracts of mesenteric arteries obtained from wild-type (WT), *gp91^{phox}* or *S1P3* knockout mice. Data are expressed as mean ± SD. Statistical analysis was performed by (**B, D** and **F**) two-way ANOVA or (**A, C, E** and **G**) two-way ANOVA RM followed by Bonferroni's *post hoc* test.

Supplemental Figure 10

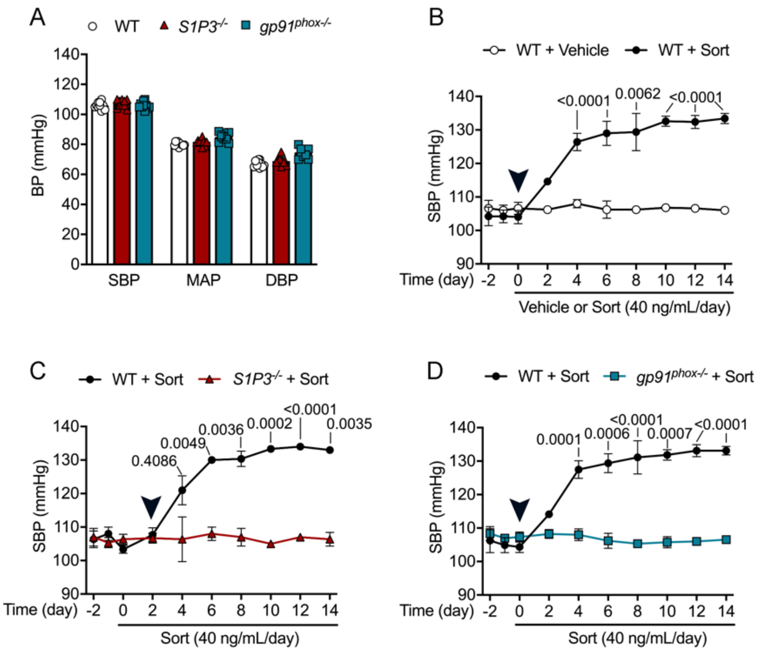

Supplemental Figure 10 Chronic sortilin administration promoted blood pressure increase in WT, but not in S1P3- or gp91^{phox}-deficient mice. (A) Baseline of systolic blood pressure (SBP), mean arterial pressure (MAP), and diastolic blood pressure (DBP) determined by tail-cuff system in WT, *S1P3*$^{-/-}$, and *gp91*$^{phox-/-}$ mice; (*n*=10). **(B,D,F)** Time courses of SBP measured before and after implantation of an osmotic pump delivering vehicle or sortilin for 14 days in **(B)** WT mice, **(D)** *S1P3*$^{-/-}$ mice, and **(F)** *gp91*$^{phox-/-}$ mice; (*n*=5-8). Arrowheads indicate the day of implantation. Results are expressed as mean ± SD. Two-way ANOVA ANOVA RM **(B,C,D)** followed by Bonferroni's *post hoc* test was used.

Supplemental Figure 11

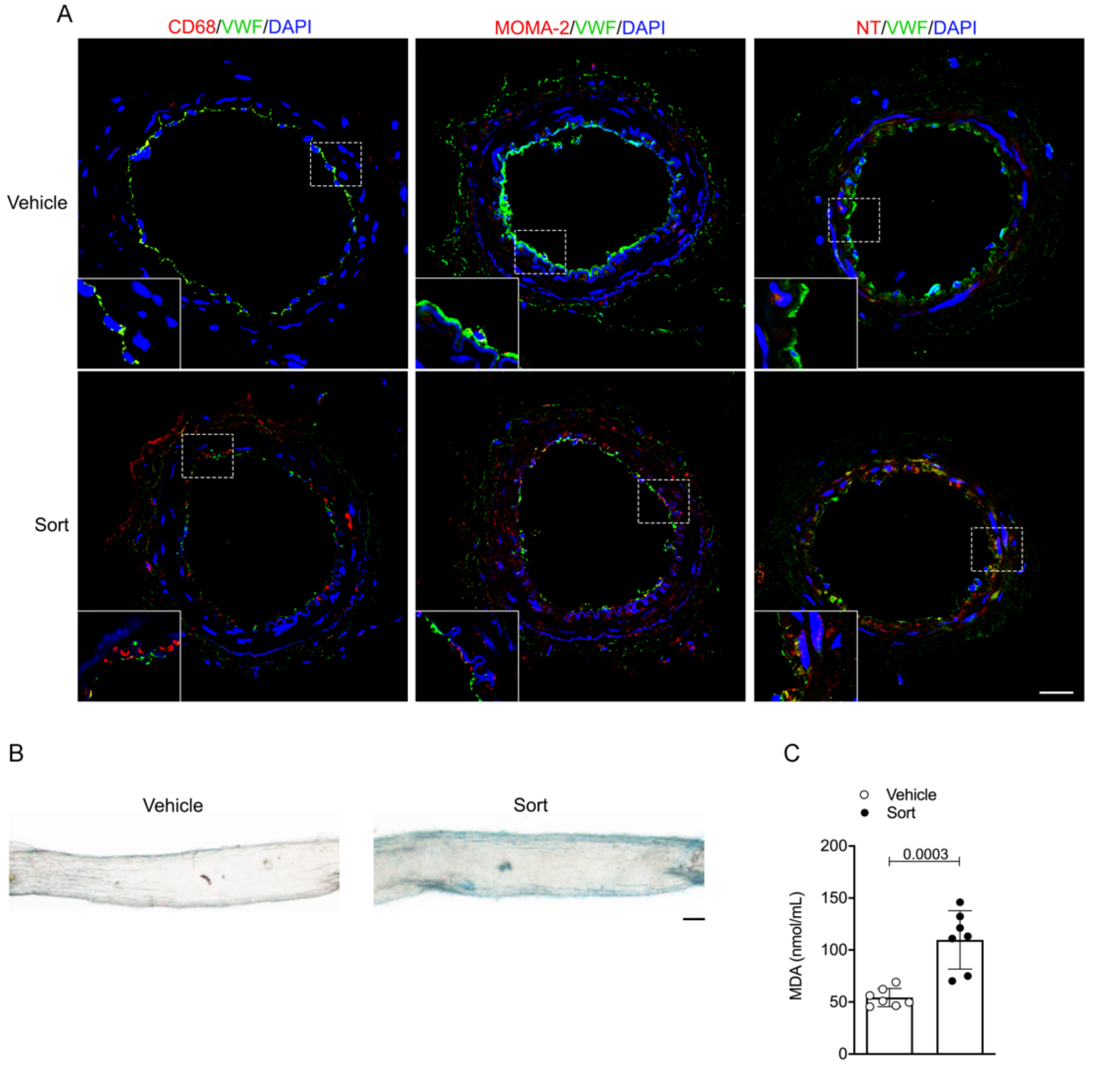

Supplemental Figure 11 Chronic sortilin treatment induces inflammation, peroxynitrite formation, and vascular permeability in mesenteric arteries. (**A**) Immunofluorescence staining of CD68, MOMA-2 and 3-nitrotyrosine (NT) in mesenteric arteries from vehicle- (top) and sortilin-treated (bottom) WT mice. Von Willebrand Factor (VWF) was used to stain the endothelium. Nuclei were stained with DAPI. Scale bar: 25 µm. (**B**) Incorporation of Evans blue dye in the vessel wall of vehicle and sortilin-exposed mesenteric arteries. The blue area corresponds to area of increased vascular permeability. Scale bar: 100 µm. (**C**) Plasma levels of malondialdehyde (MDA) levels in WT mice after chronic treatment with sortilin or vehicle; (*n*=7). Results are expressed as mean ± SD. Statistical analysis was performed by unpaired two-tailed Student’s *t*-test.

Supplemental Figure 12

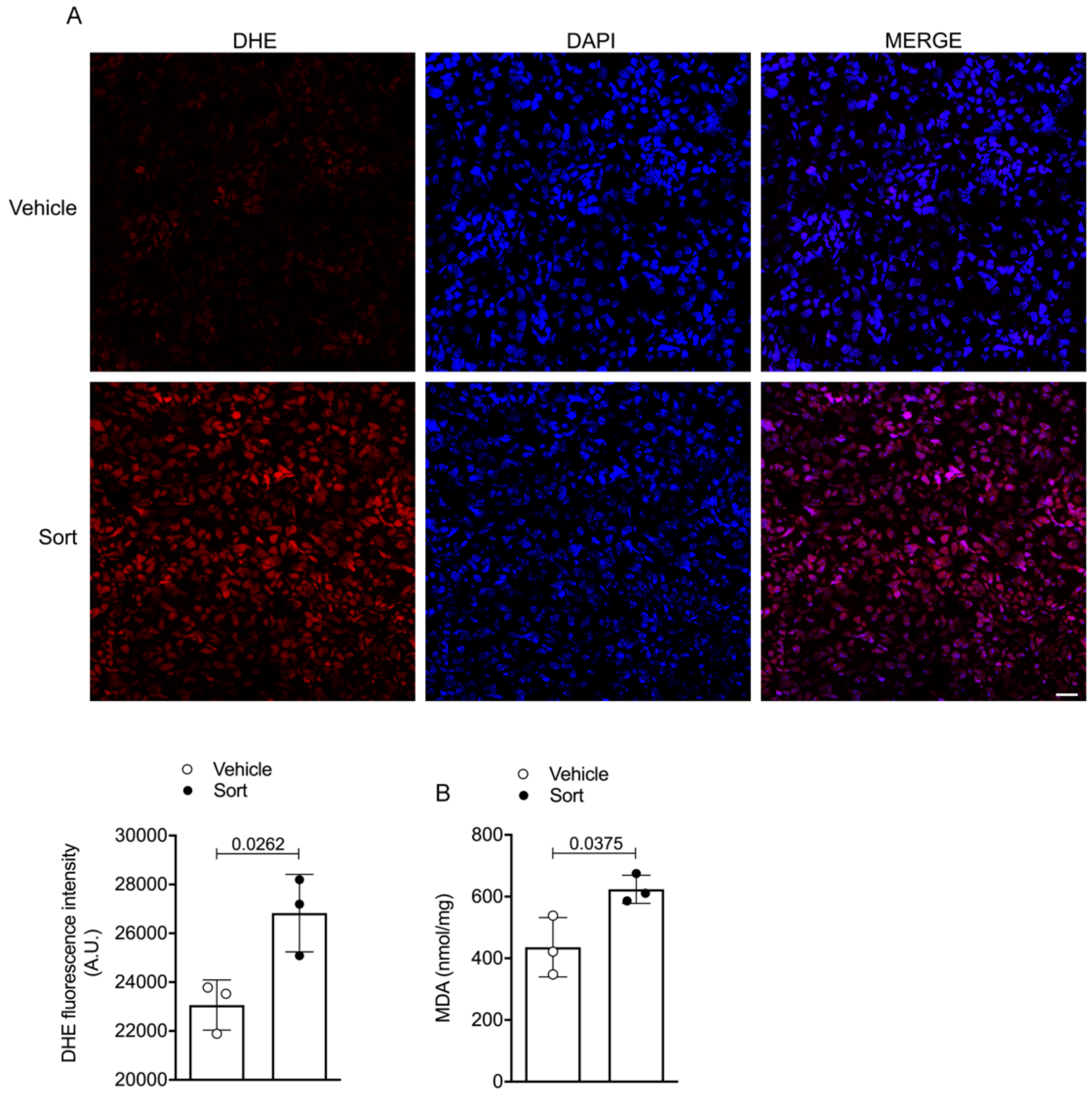

Supplemental Figure 12 Chronic sortilin treatment results in substantially increased renal oxidative stress. (**A**) Representative images of dihydroethidium (DHE) staining in kidney sections from sortilin- or vehicle-treated WT mice. Nuclei were stained with DAPI. Scale bar: 25 µm; bar graph shows quantification of DHE fluorescence intensity; (*n*=3). (**B**) Malondialdehyde (MDA) levels in kidney homogenates from WT mice treated for 14 days with vehicle or sortilin; (*n*=3). Results are expressed as mean ± SD. Statistical analysis was performed by unpaired two-tailed Student's *t*-test.

Supplemental Figure 13

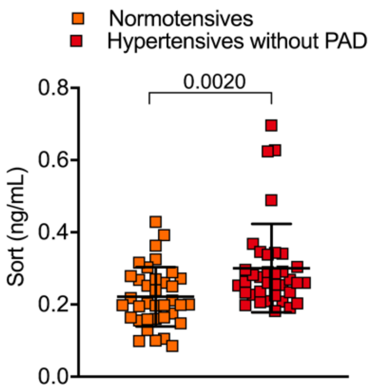

Supplemental Figure 13 Sortilin levels are elevated in hypertensive patients independently of its association with peripheral arterial disease. Plasma sortilin levels in normotensive and hypertensive subjects without peripheral arterial disease from the Campania Salute Network; (*n*=36 normotensives, *n*=36 hypertensives). Results are expressed as mean ± SD. Unpaired Student's *t*-test was used.

Supplemental Table 1. Clinical characteristics of normotensive and hypertensive individuals without PAD from the Campania Salute Network Study.

**Parameter**	**Normotensives (N=36)**	**Hypertensives without PAD (N=36)**	***P*-Value**
Clinical characteristics			
Age, years	61.8±8.8	59.1±11.7	0.264
Sex, M/F	21/15	22/14	<0.999
SBP (mmHg)	121.8±6.26	144.6±16.2	<0.0001
DBP (mmHg)	73.8±6.45	85.4±8.7	<0.0001
TC (mg/dL)	182.4±25.4	186.4±19.1	0.450
HDL (mg/dL)	56.4±12.1	53.1±10.7	0.237
TG (mg/dL)	106±28.7	116±40.1	0.2171
Glucose (mg/dL)	94.4±10.5	97.7±8.8	0.151
Creatinine (mg/dL)	0.83±0.1	0.88± 0.2	0.158
Medication (%)			
β-blockers	0	9 (25)	
RAAS-inhibitors	0	23 (63.8)	
Diuretics	0	12 (33.3)	
CCBs	0	11 (30.5)	
Statins	0	0	

SBP, systolic blood pressure; DBP, diastolic blood pressure; TC, total cholesterol; HDL, high density lipoprotein; TG, triglycerides; RAAS, Renin-angiotensin-aldosterone system inhibitors; CCBs, Calcium channel blockers. *P*-value based on unpaired Student's *t*-test for continuous variables and Fisher’s exact test for categorical variables.

Supplemental Table 2. Analysis of variance (ANOVA) results for antihypertensive drug effects on plasma sortilin levels in hypertensive patients from the Moli-Sani Study.

Uncontrolled hypertensive patients

Drugs	Sum Sq.	d.f.	Mean Sq.	F	Prob>F
Diuretics	0.02302	1	0.02302	0.91	0.3445
ACE-inhibitors	0.00004	1	0.00004	0	0.9705
CCBs	0.00008	1	0.00008	0	0.9559
β-blockers	0.00095	1	0.00095	0.04	0.8469
ARBs	0.00759	1	0.00759	0.3	0.5865
Diuretics * ACE-inhibitors	0.00032	1	0.00032	0.01	0.9109
Diuretics * CCBs	0.00063	1	0.00063	0.02	0.8757
Diuretics * β-blockers	0.05496	1	0.05496	2.16	0.1459
Diuretics * ARBs	0.00042	1	0.00042	0.02	0.8984
ACE-inhibitors * CCBs	0.02459	1	0.02459	0.97	0.3286
ACE-inhibitors * β-blockers	0.02905	1	0.02905	1.14	0.2887
ACE-inhibitors * ARBs	0.02266	1	0.02266	0.89	0.3483
CCBs * β-blockers	0.00911	1	0.00911	0.36	0.5513
CCBs * ARBs	0.00123	1	0.00123	0.05	0.8265
β-blockers * ARBs	0.00057	1	0.00057	0.02	0.8813
Diuretics * ACE-inhibitors * CCBs	0.09549	1	0.09549	3.76	0.0566
Diuretics * ACE-inhibitors * β-blockers	0.05173	1	0.05173	2.04	0.1581
Diuretics * ACE-inhibitors * ARBs	0.00435	1	0.00435	0.17	0.6803
# Diuretics * CCBs * β-blockers	0	0	0	0	NaN
# Diuretics * CCBs * ARBs	0	0	0	0	NaN
# Diuretics * β-blockers * ARBs	0	0	0	0	NaN
# ACE-inhibitors * CCBs * β-blockers	0	0	0	0	NaN
# ACE-inhibitors * CCBs * ARBs	0	0	0	0	NaN
# ACE-inhibitors * β-blockers * ARBs	0	0	0	0	NaN
# CCBs * β-blockers * ARBs	0	0	0	0	NaN
Error	1.80471	71	0.02542		
Total	2.13149	89			

CCBs, Calcium channel blockers; ARBs, Angiotensin II receptor blockers. Terms marked with # are not full rank.

Controlled hypertensive patients

Drugs	Sum Sq.	d.f.	Mean Sq.	F	Prob>F
Diuretics	0.0068	1	0.0068	0.53	0.4708
ACE-inhibitors	0.00087	1	0.00087	0.07	0.7965
CCBs	0.02446	1	0.02446	1.89	0.1734
β-blockers	0.02201	1	0.02201	1.7	0.1963
ARBs	0.00042	1	0.00042	0.03	0.8568
Diuretics * ACE-inhibitors	0.00201	1	0.00201	0.16	0.6947
Diuretics * CCBs	0.00178	1	0.00178	0.14	0.712
Diuretics * β-blockers	0.00228	1	0.00228	0.18	0.6758
Diuretics * ARBs	0.00045	1	0.00045	0.04	0.8519
ACE-inhibitors * CCBs	0.00654	1	0.00654	0.5	0.4795
ACE-inhibitors * β-blockers	0.00002	1	0.00002	0	0.967
# ACE-inhibitors * ARBs	0	0	0	0	NaN
CCBs * β-blockers	0.00071	1	0.00071	0.5	0.8153
CCBs * ARBs	0.00683	1	0.00683	0.53	0.4698
β-blockers * ARBs	0.00761	1	0.00761	0.59	0.4455
# Diuretics * ACE-inhibitors * CCBs	0	0	0	0	NaN
Diuretics * ACE-inhibitors * β-blockers	0.00771	1	0.00771	0.6	0.4426
# Diuretics * ACE-inhibitors * ARBs	0	0	0	0	NaN
# Diuretics * CCBs * β-blockers	0	0	0	0	NaN
# Diuretics * CCBs * ARBs	0	0	0	0	NaN
Diuretics * β-blockers * ARBs	0.02393	0	0.02393	1.85	0.1781
# ACE-inhibitors * CCBs * β-blockers	0	0	0	0	NaN
# ACE-inhibitors * CCBs * ARBs	0	0	0	0	NaN
# ACE-inhibitors * β-blockers * ARBs	0	0	0	0	NaN
# CCBs * β-blockers * ARBs	0	0	0	0	NaN
Error	0.95799	74	0.01295		
Total	1.09979	90			

CCBs, Calcium channel blockers; ARBs, Angiotensin II receptor blockers. Terms marked with # are not full rank.

Supplemental Table 3. Details of primers used for RT-qPCR.

Target gene	Primer Sequence	Amplicon size (bp)	T_A	GenBank Acc. No.
S1P3	Forward 5′- TGATTGTGGTGAGCGTGTTCA-3′<br>Reverse 5′- GGCCACATCAATGAGGAAGAG-3′	68	60°C	NM_005226
NOX2	Forward 5′-TGGATAGTGGGTCCCATGTT-3′<br>Reverse 5′-GCTTATCACAGCCACAAGCA-3′	307	58°C	NM_000397
NOX4	Forward 5′-CCGGCTGCATCAGTCTTAACC-3′<br>Reverse 5′-TCGGCACAGTACAGGCACAA-3′	220	61°C	NM_016931
GAPDH	Forward 5′-AAGGTGAAGGTCGGAGTCAA-3′<br>Reverse 5′-AATGAAGGGGTCATTGATGG-3′	108	58°C	NM_002046

T_A, annealing temperature.

Full unedited gels for Figure 1C

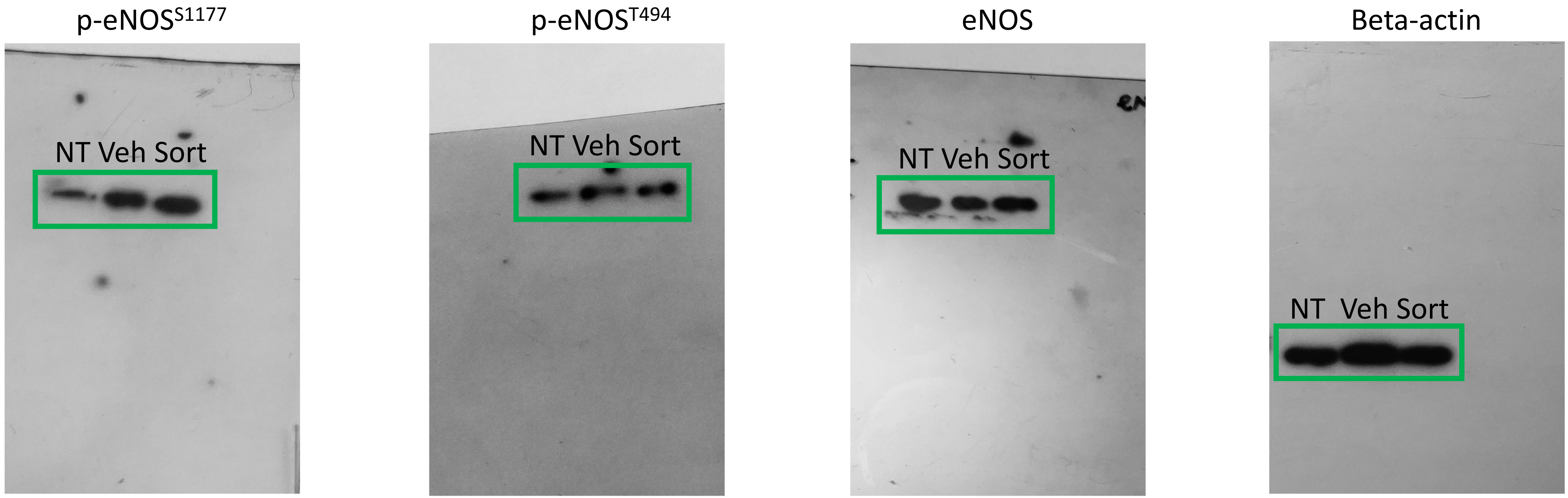

The lanes in green box correspond to those shown in the manuscript

Full unedited gels for Figure 2C

ASMase

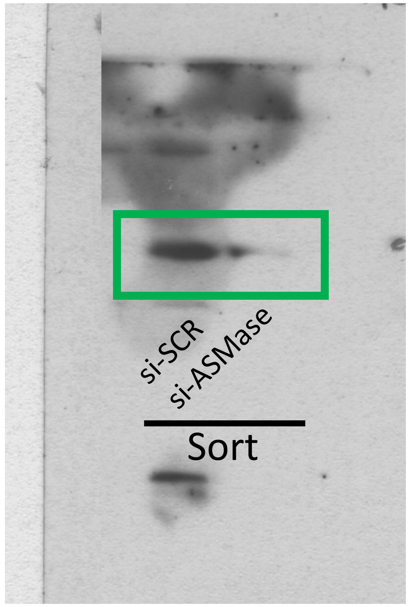

Beta-actin

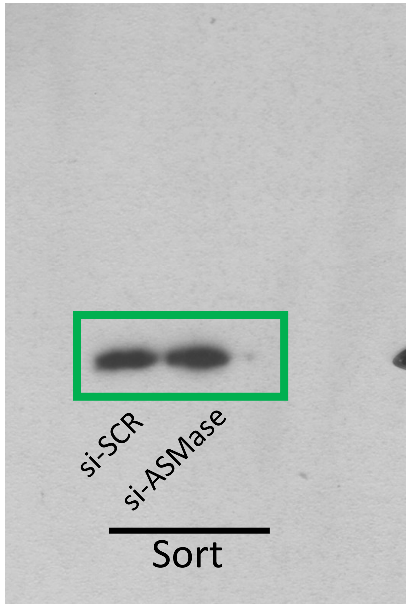

The lanes in green box correspond to those shown in the manuscript

Full unedited gels for Figure 2H

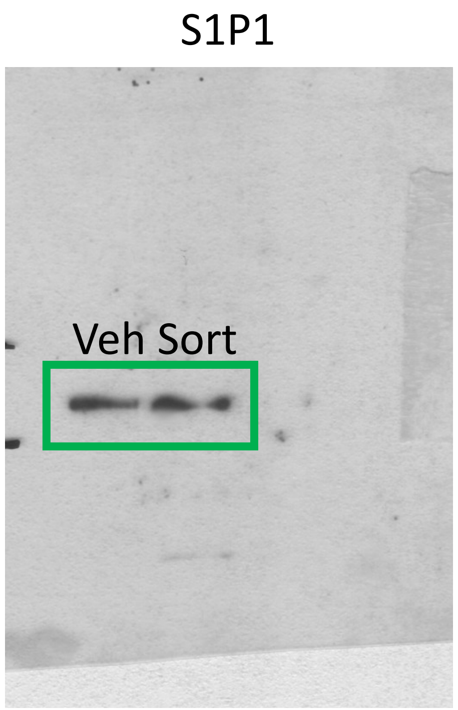

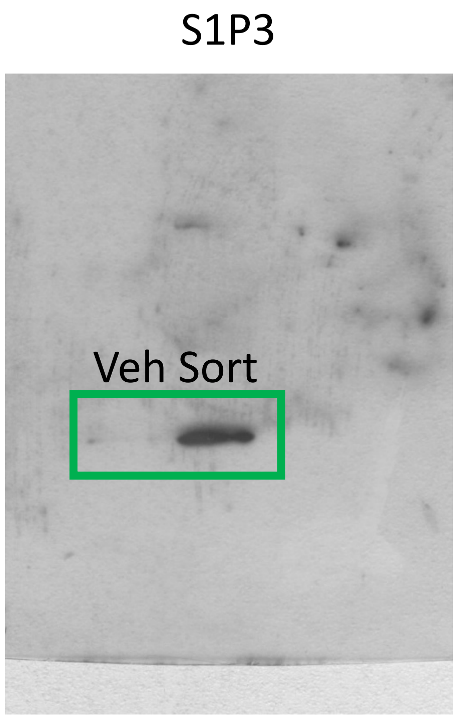

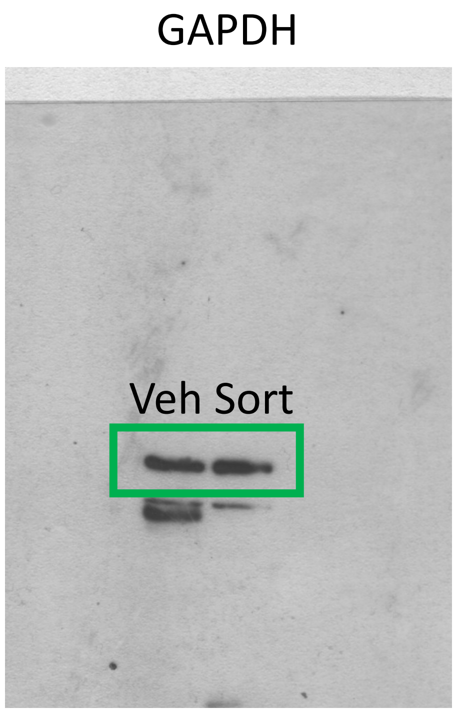

The lanes in green box correspond to those shown in the manuscript

Full unedited gels for Figure 3G

p-PYK2^{Y579}

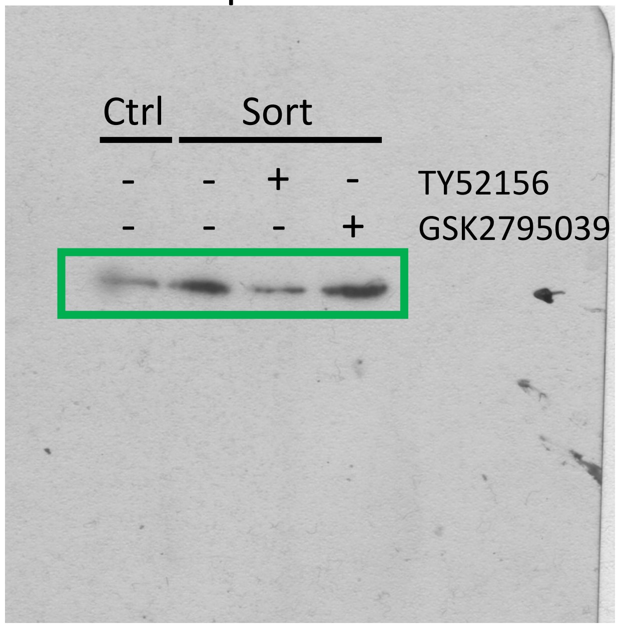

p-PKCε^{S729}

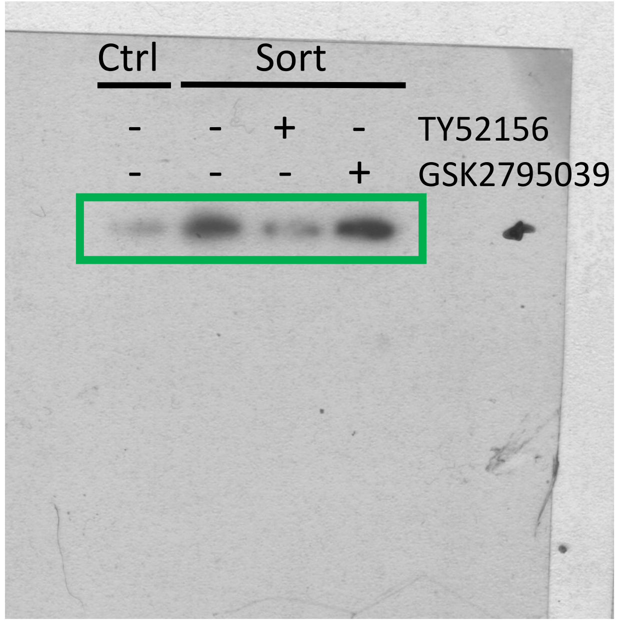

p-PKCα^{T497}

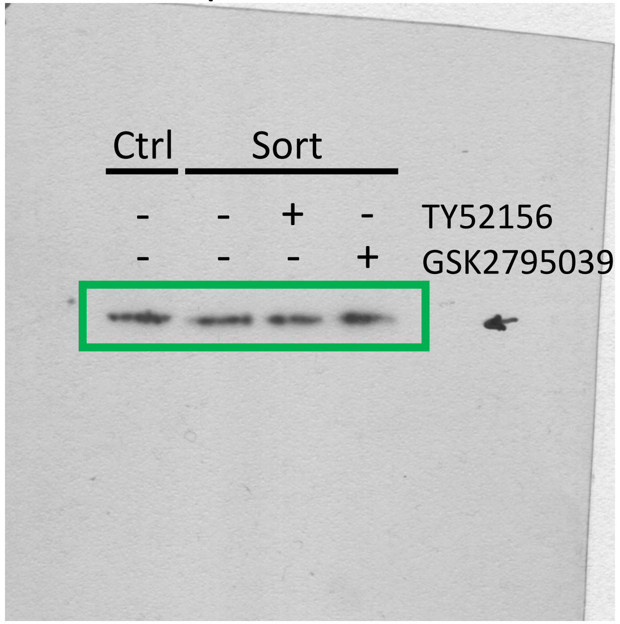

PKC

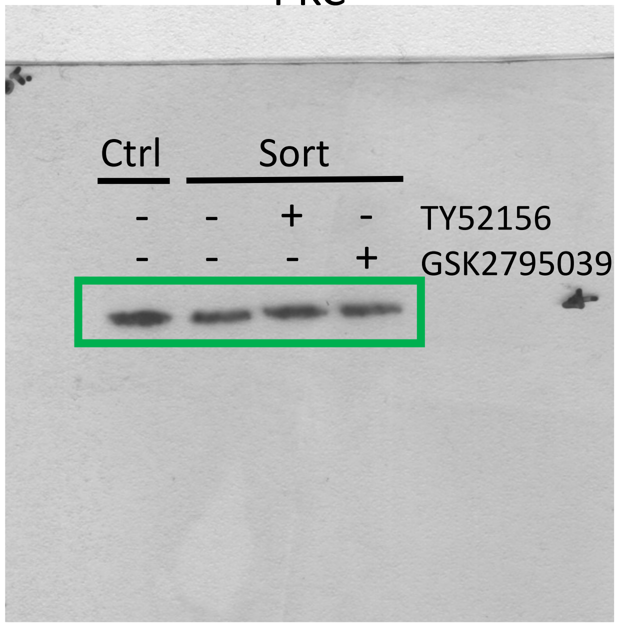

The lanes in green box correspond to those shown in the manuscript

Full unedited gels for Figure 3G

Beta-actin

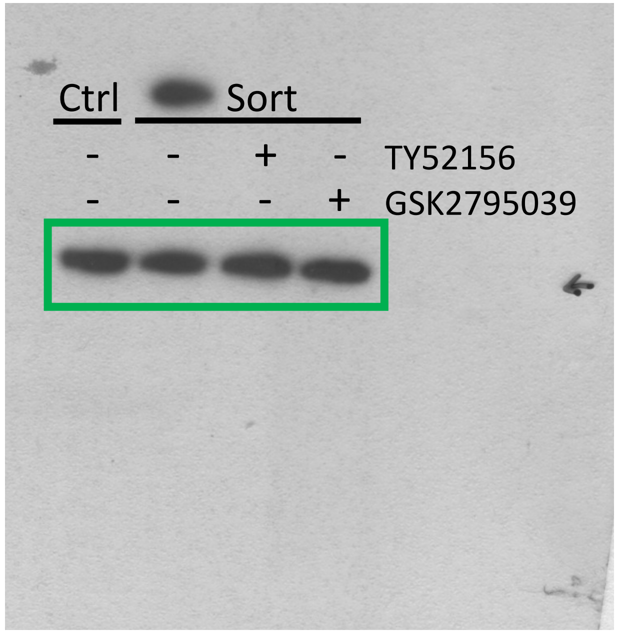

S1P3

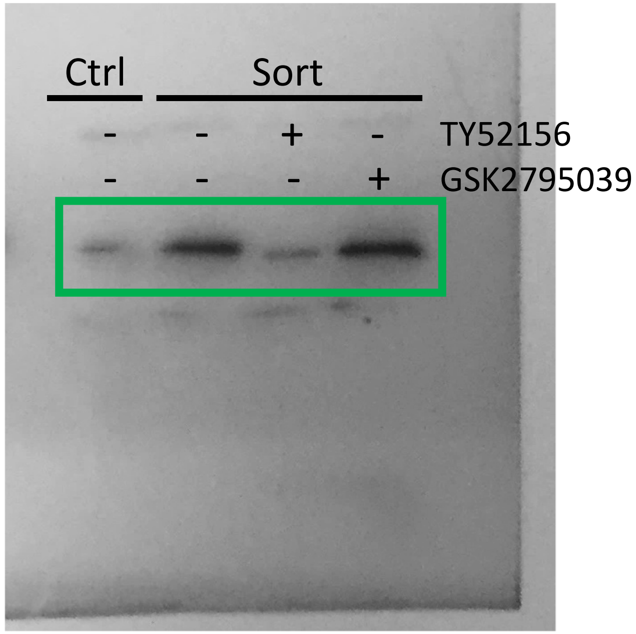

Rac1-GTP

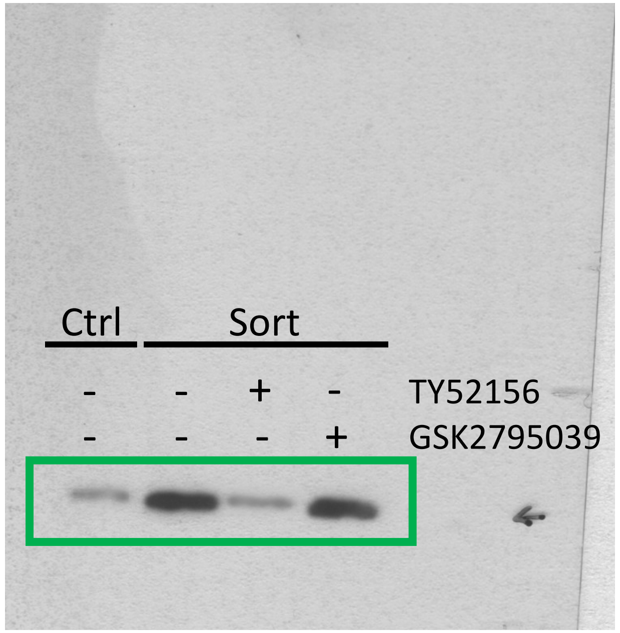

The lanes in green box correspond to those shown in the manuscript

Full unedited gels for Figure 4A

p-PYK2^{Y579}

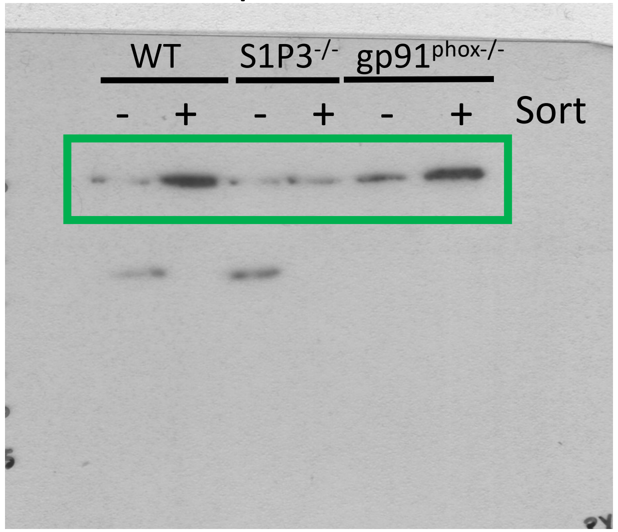

p-PKCε^{S729}

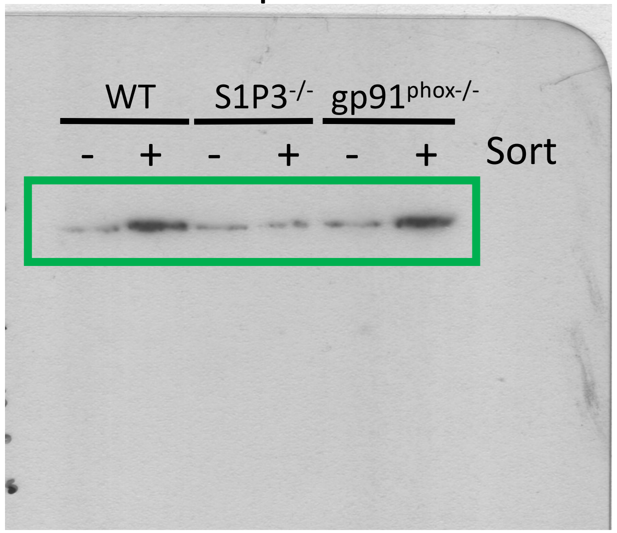

PKC

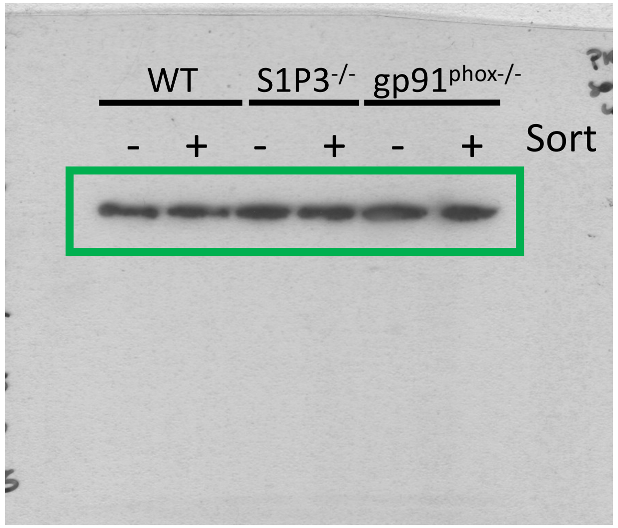

Beta-actin

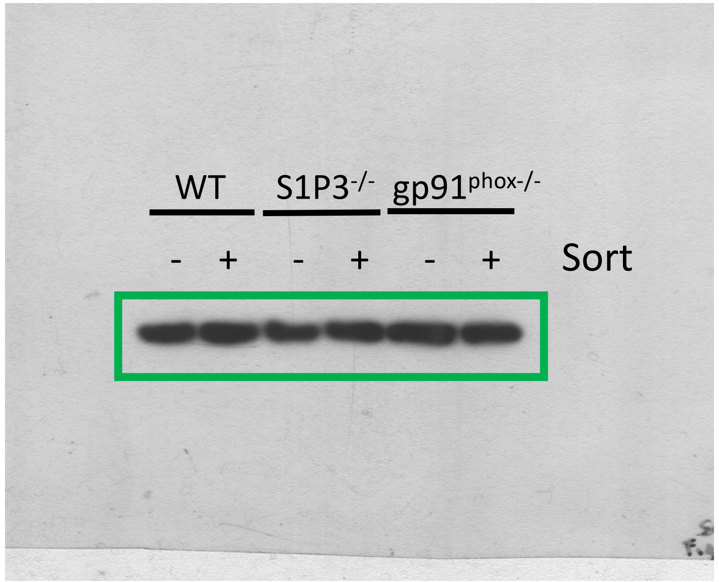

Rac1-GTP

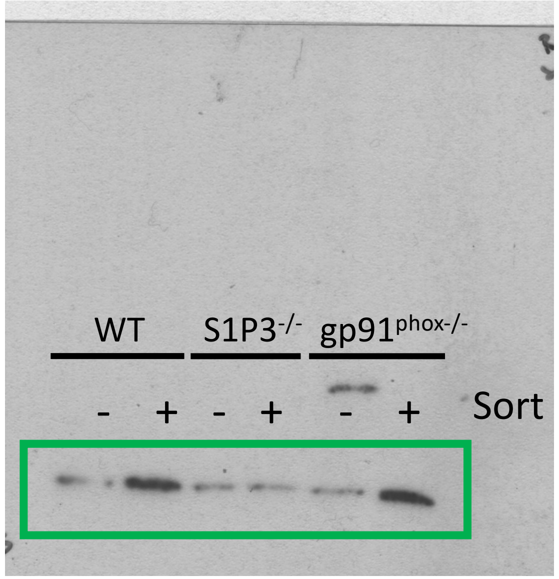

The lanes in green box correspond to those shown in the manuscript

Full unedited gels for Figure 4C

p-PYK2^{Y579}

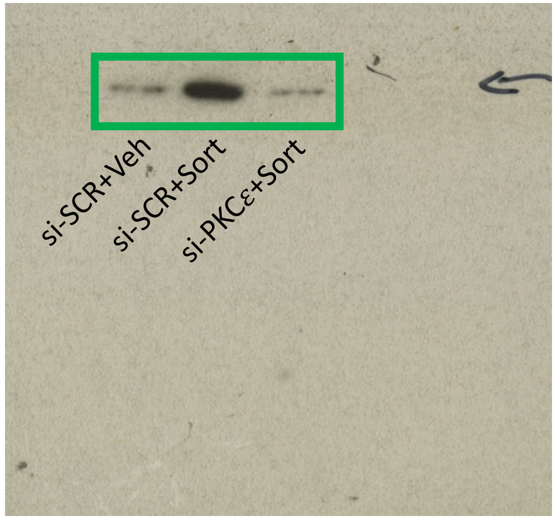

Beta-actin

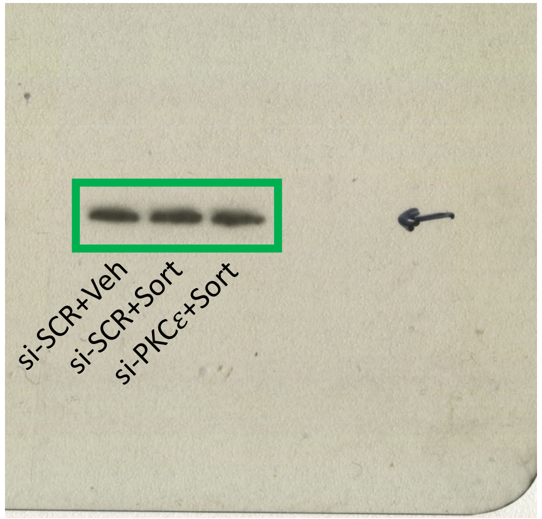

PKCε

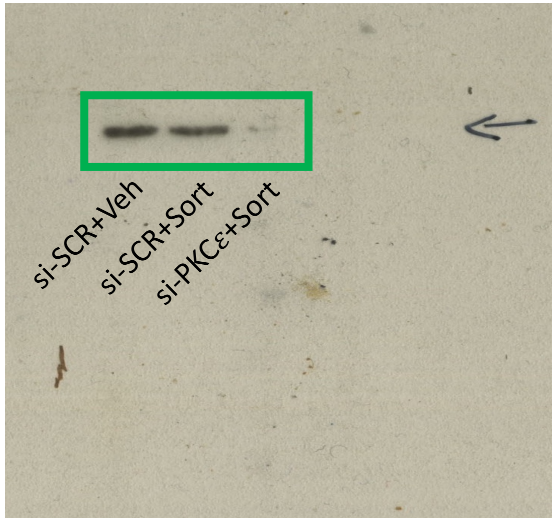

Rac1-GTP

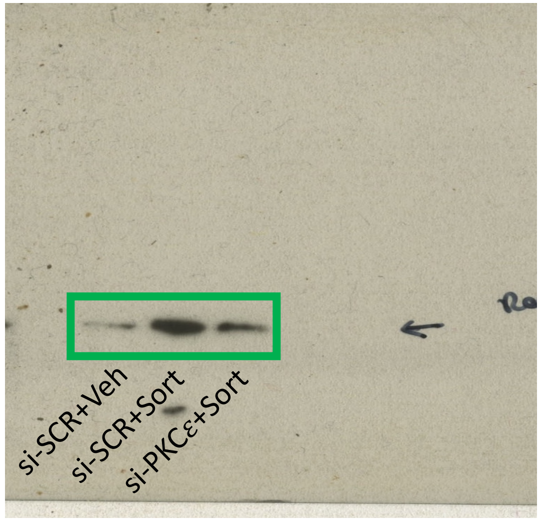

The lanes in green box correspond to those shown in the manuscript

Full unedited gels for Supplemental Figure 2A

aCDase

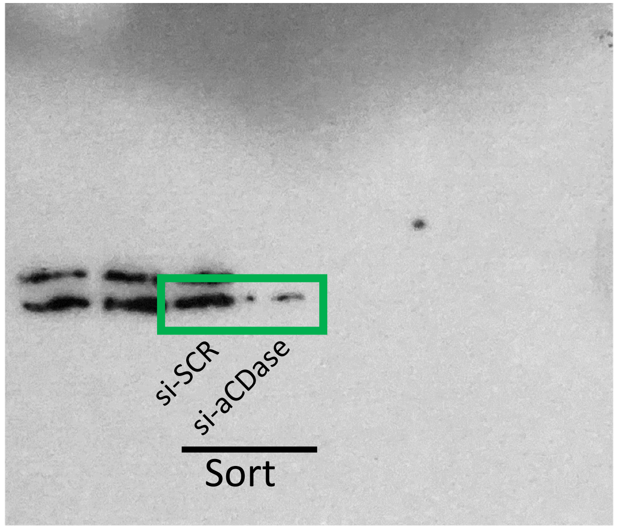

GAPDH

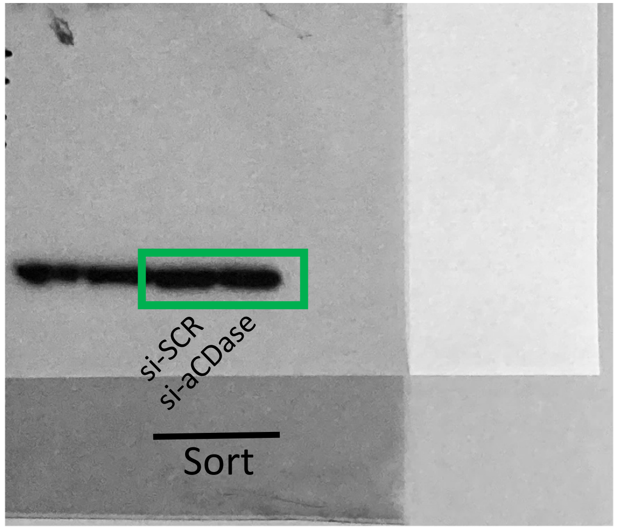

The lanes in green box correspond to those shown in the manuscript

Full unedited gels for Supplemental Figure 2B

SphK1

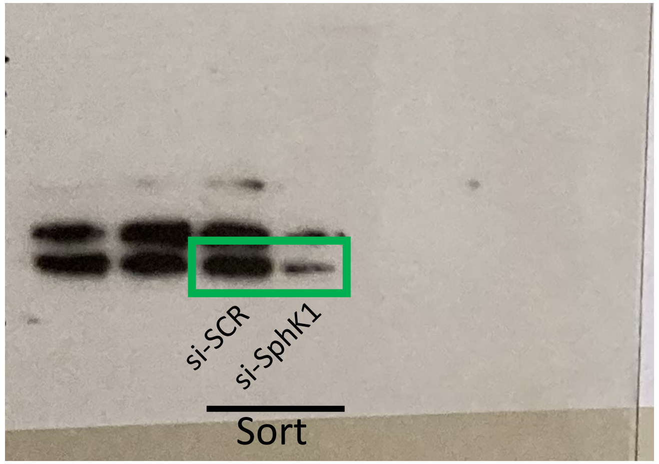

GAPDH

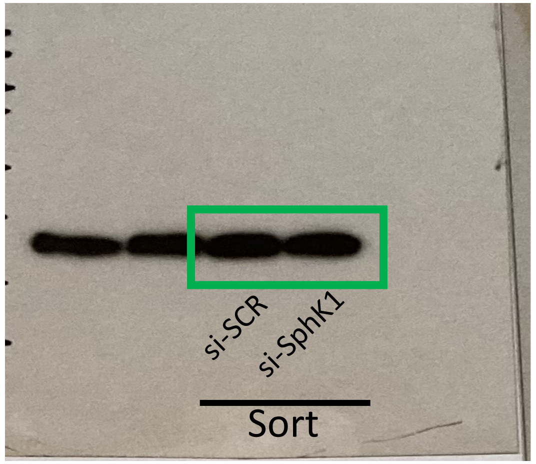

The lanes in green box correspond to those shown in the manuscript

Full unedited gels for Supplemental Figure 2C

SphK2

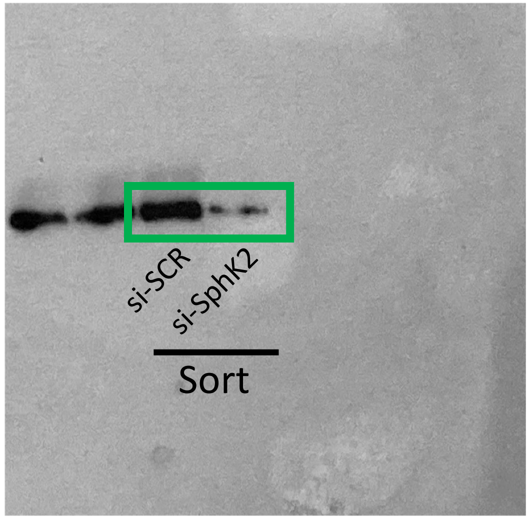

GAPDH

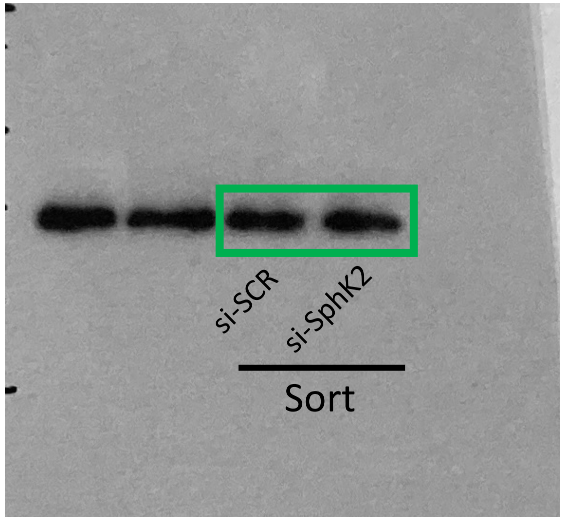

The lanes in green box correspond to those shown in the manuscript

Full unedited gels for Supplemental Figure 5A

S1P1

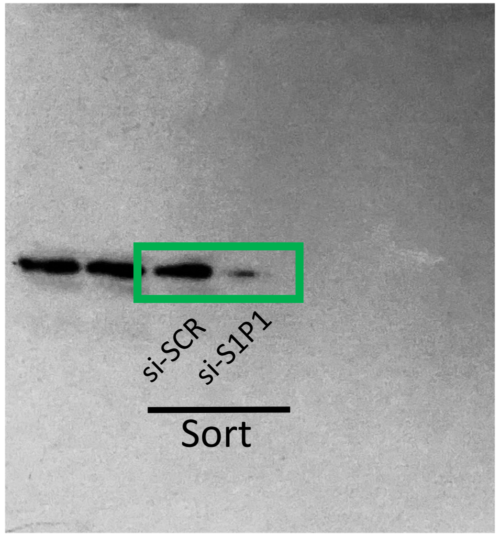

GAPDH

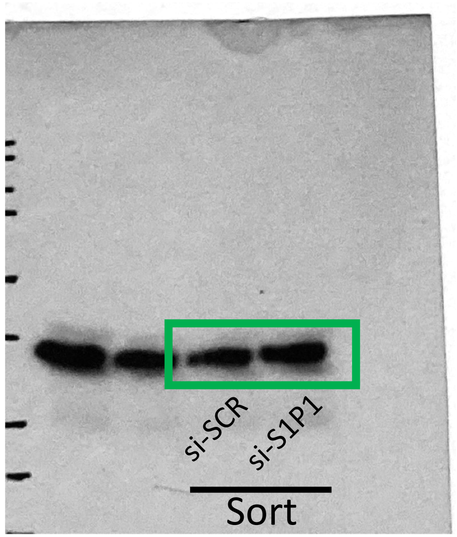

The lanes in green box correspond to those shown in the manuscript

Full unedited gels for Supplemental Figure 5B

S1P3

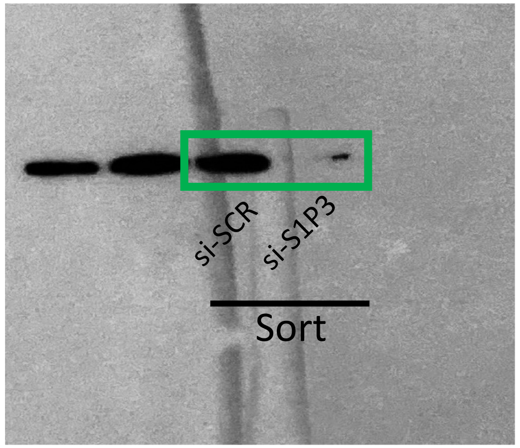

GAPDH

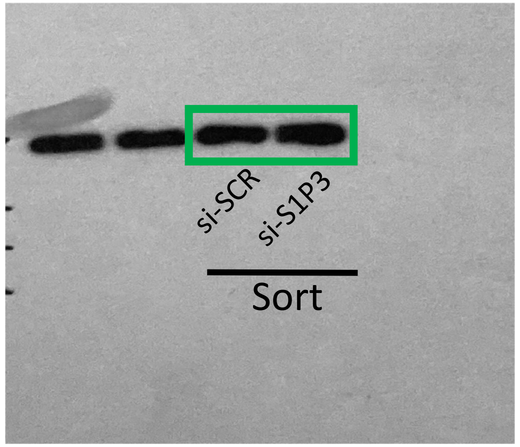

The lanes in green box correspond to those shown in the manuscript

Full unedited gels for Supplemental Figure 9H

$gp91^{phox}$

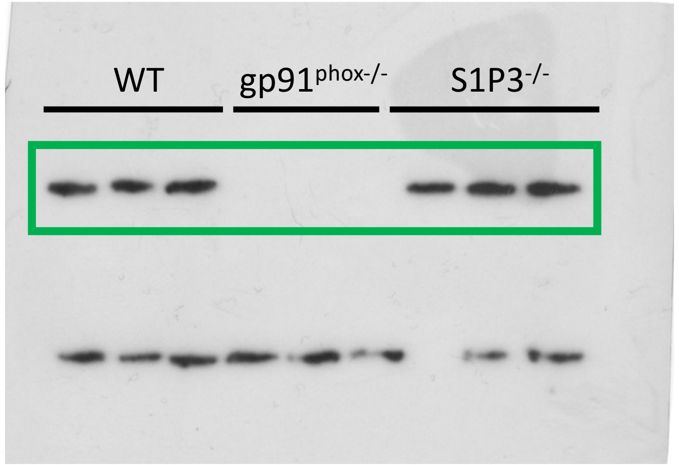

S1P3

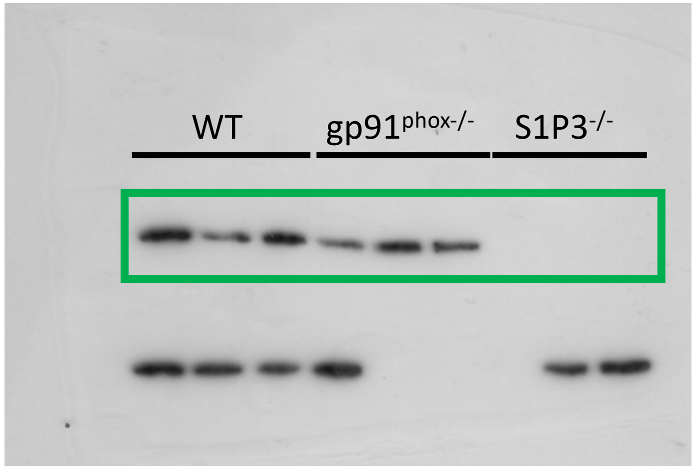

GAPDH

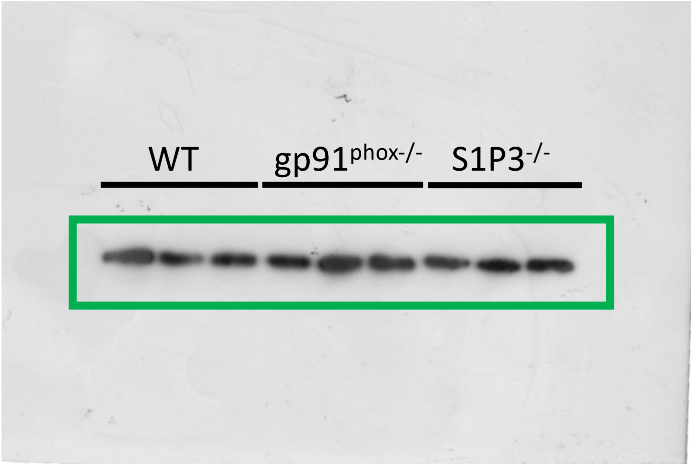

The lanes in green box correspond to those shown in the manuscript
